# A Comprehensive Review on Sustainable Triboelectric Energy Harvesting Using Biowaste-Derived Materials

**DOI:** 10.3390/ma19030592

**Published:** 2026-02-03

**Authors:** Wajid Ali, Tabinda Shabir, Shahzad Iqbal, Syed Adil Sardar, Farhan Akhtar, Woo Young Kim

**Affiliations:** Department of Electronic Engineering, Jeju National University, Jeju 63243, Republic of Korea

**Keywords:** triboelectric nanogenerators (TENGs), biowaste-derived materials, sustainable energy harvesting, waste valorization, surface engineering, material processing, biodegradable, biocompatible materials

## Abstract

The growing demand for sustainable and distributed energy solutions has driven increasing interest in triboelectric nanogenerators (TENGs) as platforms for energy harvesting and self-powered sensing. Biowaste-based triboelectric nanogenerators (BW-TENGs) represent an attractive strategy by coupling renewable energy generation with waste valorization under the principles of the circular bioeconomy. This review provides a comprehensive overview of BW-TENGs, encompassing fundamental triboelectric mechanisms, material categories, processing and surface-engineering strategies, device architectures, and performance evaluation metrics. A broad spectrum of biowaste resources—including agricultural residues, food and marine waste, medical plastics, pharmaceutical waste, and plant biomass—is critically assessed in terms of physicochemical properties, triboelectric behavior, biodegradability, biocompatibility, and scalability. Recent advances demonstrate that BW-TENGs can achieve electrical outputs comparable to conventional synthetic polymer TENGs while offering additional advantages such as environmental sustainability, mechanical compliance, and multifunctionality. Key application areas, including environmental monitoring, smart agriculture, wearable and implantable bioelectronics, IoT networks, and waste management systems, are highlighted. The review also discusses major challenges limiting large-scale deployment, such as material heterogeneity, environmental stability, durability, and lack of standardization, and outlines emerging solutions involving material engineering, hybrid energy-harvesting architectures, artificial intelligence-assisted optimization, and life cycle assessment frameworks.

## 1. Introduction

The increasing rate of energy demands in the world, accompanied by the growing degree of climate change and environmental degradation, requires the development of sustainable and environmentally friendly energy solutions [1,2,3]. Worldwide energy consumption grew by some 2.2% in 2024 (above historical averages of 1.5% (201,019 on average)). Electricity demand rose by 4.2%, equivalent to 1.12 TWh, among the highest absolute growth rates ever [4,5]. This surge reflects rapid industrial expansion in developing economies (with BRICS countries accounting for nearly half of global electricity-processing capacity and experiencing ~5% annual growth), record-breaking temperatures that have increased cooling demand, the rapid expansion of AI and data center infrastructure consuming approximately 240–340 TWh annually, and global electric vehicle sales exceeding 20 million units [6,7]. Although renewable sources—solar, wind, and hydropower—generated a record 858 TWh of electricity (40.9% of global electricity supply, the highest share since the 1940s), fossil fuel generation continued to rise to meet peak loads [8]. This highlights a fundamental limitation of conventional centralized power systems: they are increasingly unable to meet the diverse energy requirements of distributed, portable, and autonomous electronic systems operating without direct grid access.

### 1.1. Limitations of Conventional Batteries in Wearable and IoT Systems

The widespread adoption of wearable and Internet of Things (IoT) devices has led to rapidly increasing power demands. Conventional chemical batteries impose fundamental limitations on device miniaturization, operational lifetime, and overall sustainability. Their constrained energy density (typically 150–250 Wh/kg for lithium ion batteries, approaching thermodynamic limits), susceptibility to thermal runaway and associated thermal management challenges, and the need for frequent recharging (daily or weekly for smartwatches and fitness trackers) undermine device reliability and user acceptance. These limitations are particularly critical for applications requiring continuous and long-term health monitoring [9]. Medical-grade wearables, such as ECG patches and continuous glucose monitors, require uninterrupted power supply but are unable to operate for more than 30 days in continuous mode due to prohibitive size and weight constraints, which prevent practical on-skin integration or implantation. Similarly, IoT applications deployed in inaccessible or remote environments, including environmental monitoring systems, agricultural sensors, and underground sensing networks, demand long-term, maintenance-free operation, where battery replacement is either economically unfeasible or physically impractical. Consequently, the realization of an IoT-enabled smart world, encompassing smart cities, precision agriculture, and advanced healthcare delivery, critically depends on technologies capable of harvesting ambient energy without relying on consumable chemical energy storage. Such approaches would enable truly autonomous system operation over timescales of years to decades [10,11].

### 1.2. Triboelectric Nanogenerators: Working Principles, Operating Modes, and Performance Advantages

**Figure 1:**
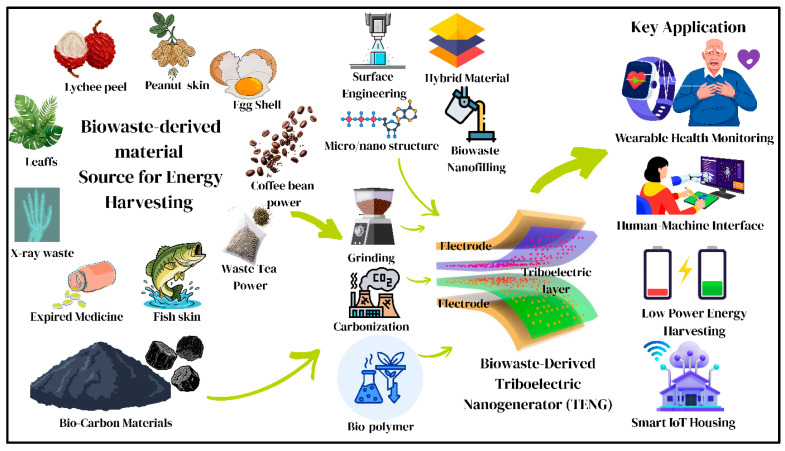
Overview of biowaste-derived triboelectric nanogenerators (BW-TENGs). Diverse biowaste sources are processed into active triboelectric materials and integrated into TENGs for mechanical energy harvesting. Key applications include wearable electronics, human–machine interfaces, low-power energy harvesting, and smart IoT systems.

Since their introduction in 2012, triboelectric nanogenerators (TENGs) have emerged as a promising technology for converting ubiquitous low-frequency mechanical energy such as human motion, wind, ocean waves, structural vibrations, and rainfall into electrical energy [12,13,14,15]. TENGs operate based on contact electrification and electrostatic induction, enabling the harvesting of mechanical energy over an exceptionally broad frequency range, from <0.1 Hz (e.g., human gait and heartbeat) to >10 kHz (e.g., machinery vibrations) [12,16,17]. Notably, in low-frequency regimes, TENGs outperform conventional inertial microgenerators and piezoelectric devices. Compared with traditional energy harvesters, including photovoltaic, electromagnetic, and piezoelectric systems, TENGs offer several distinct advantages [18]. They can employ a wide variety of materials, as nearly any dielectric polymer, biopolymer, or carbon-based material can function as a triboelectric layer. Their device architectures are simple, often based on planar contact–separation configurations, and they can be fabricated at low cost (typically below 1–10 USD/kg of material) [19]. In addition, TENGs exhibit high energy conversion efficiencies, generally in the range of 50–85%. With reported real power densities reaching up to ~1200 W/m^2^ and volumetric power densities as high as ~490 kW/m^3^, TENGs rank among the most efficient mechanical energy-harvesting technologies currently available [20]. TENG operation is generally categorized into four fundamental working modes: contact–separation, which generates pulsed outputs through periodic contact under reciprocating motion; lateral sliding, which enables continuous energy harvesting through tangential motion such as walking; single-electrode mode, which simplifies device configuration; and freestanding mode, in which triboelectric layers can flutter under wind or fluid flow without rigid support structures [21]. Contact electrification governed by charge transfer between materials with differing electronegativities operates through the electron cloud–potential-well model. Upon intimate atomic-scale contact, overlapping electron clouds generate interfacial dipoles that drive electron transfer until Fermi-level equilibrium is established. Upon separation, the retained surface charges create electrostatic fields that induce macroscopic current flow through an external circuit [22]. Under high-humidity or aqueous conditions, ion-transfer mechanisms (e.g., H^+^ and OH^−^) may also contribute, providing additional charge-transfer pathways. Repeated contact–separation cycles generate alternating electrical outputs (typically 10–1000 V with instantaneous power levels of 1–100 mW), enabling continuous energy conversion from low-frequency mechanical motions. Despite their impressive performance, most conventional TENGs rely on petroleum-derived, non-biodegradable polymers such as PTFE, PDMS, PET, and nylon, which exhibit environmental persistence on century-long timescales. Although these materials are highly effective as triboelectric layers, their synthetic origin and poor degradability introduce a significant sustainability paradox [23]. Less than 10% of the more than 400 million tons of polymers produced annually is recycled, while the remaining ~90% accumulates in landfills (exceeding 650 million tons) or disperses into natural ecosystems. Consequently, microplastics have been detected everywhere from Arctic permafrost to deep ocean sediments, with growing evidence of toxicological impacts on both ecosystems and human health. This contradiction, advancing high-performance energy-harvesting technologies while simultaneously exacerbating non-degradable waste accumulation, highlights the urgent need for alternative material strategies [24]. Addressing this challenge motivates the exploration of sustainable and regenerative materials that can deliver efficient energy harvesting while minimizing environmental burden, marking a paradigm shift away from petroleum dependence toward waste-valorization approaches aligned with circular bioeconomy principles [25]. The comprehensive overview is shown in Figure 1 of biowaste material from different waste energizing low-powered devices by applying different approaches to make proper use of it.

Biowaste refers to plant and animal materials of agricultural, household, food, and industrially generated origin, which naturally disintegrate in weeks to years. Dispersed sources such as agricultural waste (representative streams: agricultural waste > 1.5 billion tons a year: cereal straw, corn husks, bagasse, rice husks; food/household waste > 1.3 billion tons a year: fruit/vegetable peels, coffee waste, egg shells, animal bones; marine waste > 40 million tons a year: fish scales, mollusk shells, seaweed; bio-carbon sources > 100 million tons a year: biochar, activated carbon, hydrochar) are low-cost (negative cost due to the use of waste disposal avoidance), biocompatible (implantable/wearable, not cytotoxic), and biodegradable (months to years in decomposition), and these materials are available worldwide (global availability), solving the waste management and energy problems at the same time [26,27].

### 1.3. Physicochemical and Structural Advantages of Biowaste for TENGs

Importantly, biowaste materials are not just substitutes that are environmentally benign, and they do have inherent physicochemical characteristics that are more conducive to triboelectric harvesting. Chemistry on the rich surface (vast amounts of –OH, –COOH, –NH_2_, –CONH) facilitates transfer of charges by hydrogen bonding and dipole interactions, producing higher output than inert hydrocarbon surfaces [28,29,30]. Hierarchical microarchitectures (cellulose microfibrils, mineralized matrices, collagen triple helices) expand the effective contact area and locations of charge generation in comparison to smooth synthetic polymers. Biowaste-based TENGs can be made with exceptional mechanical compliance to conform to curved surfaces and remain in contact with a strain of over 30% without loss of performance, unlike brittle synthetics. A number of natural biopolymers (collagen, chitin, cellulose) have an intrinsic piezoelectricity (1–5 pC/N), which adds to the generation of charges using both triboelectric and piezoelectric processes. Recent experiments show that biowaste-based TENGs can reach the same or even higher outputs than conventional TENGs [31]. Plant-skin TENGs (bamboo [32], onion [33], watermelon [34]) produce 150–800 V at 0.5–8 W/m^2^, cellulose/chitosan systems produce 50–200 V at 50–100 mW/m^2^ [35,36], and carbonized biomass can produce hybrid TENGs with an outputs of 100–255 mW/m^2^ and can also store energy [37,38,39], and marine biowastes (fish scales, eggshells, shell powder) are likewise effective [40,41,42]. Biowaste-derived TENGs (BW-TENGs) find applications in wearable electronics, biomedical sensors, and distributed energy harvesting for smart city systems, where compliance, adaptability, biocompatibility, and scalable material systems are paramount [43]. The given review thoroughly evaluates triboelectric energy harvesting based on biowaste-derived materials, including the underlying mechanisms, material classification and properties, processing and surface engineering approaches, device design, and applications in self-powered sensing, wearable and implantable bioelectronics, environmental monitoring, smart agriculture, and waste management within the framework of the circular economy.

This review gives an overview of triboelectric nanogenerators (BW-TENGs) derived from biowaste in all aspects, covering the underlying triboelectric principles behind the generation, material engineering-based approaches, and the design of the devices in a sustainable energy-harvesting fashion. The discussed biowaste materials are agricultural residues (straw, husks, bagasse, and plant skins), food and household wastes (peels of fruits and vegetables, coffee waste, and eggshells), marine biowastes (fish scales, mollusk shells, shell powders), biopolymer-based wastes (cellulose, chitin, chitosan, and collagen), and bio-carbon materials (biochar, activated carbon, hydrochar, and carbonized biomass). The review also looks at the preparation and surface-engineering pathways, the performance features, and the working modes of TENGs, and finally carries out a discussion of the uses in wearable and implantable electronics, self-powered sensors, smart agriculture, environmental monitoring, and Internet of Things systems. Lastly, a discussion of current challenges, sustainability implications, and future research directions is carried out in order to place BW-TENGs in a circular economy context in long-term, autonomous, and self-powered electronic systems.

## 2. Fundamentals of Triboelectric Nanogenerators

Triboelectric nanogenerators (TENGs) are mechanical energy-harvesting systems capable of converting low-frequency mechanical stimuli into electrical energy through the combined effects of contact electrification and electrostatic induction. Since their introduction in 2012, TENGs have attracted considerable attention due to their high energy conversion efficiency, broad material compatibility, simple structural design, and scalability [44]. A clear understanding of charge generation mechanisms, operating modes, and performance evaluation methods is essential for rational material selection and device optimization, particularly when extending TENG technology to biowaste-derived materials. Contact electrification (also termed triboelectrification, CE) is a ubiquitous physical phenomenon in which electrical charges are generated on material surfaces following physical contact and subsequent separation [45]. When two materials with dissimilar electron affinity come into contact, interfacial charge transfer occurs, leading to the accumulation of opposite charges on their surfaces. Upon separation, these charges remain spatially separated, generating an electric field across the interface. This electric field drives electron flow through an external circuit via electrostatic induction, enabling the conversion of mechanical motion into electrical energy [46]. The triboelectric series classifies materials according to their tendency to gain or lose electrons, with materials toward the negative end preferentially gaining electrons and those toward the positive end tending to lose electrons.

**Figure 2:**
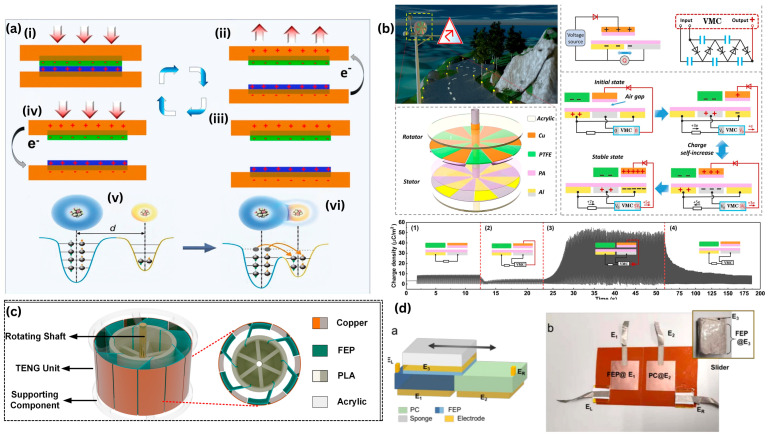
Fundamental working principles, charge transfer mechanisms, and representative device architectures of triboelectric nanogenerators (TENGs). (**a**) Schematic illustration of contact electrification and electrostatic induction processes during repeated contact–separation cycles, including the electron cloud–potential well model governing interfacial charge transfer [56]. (**b**) Representative TENG working modes and charge transport behavior under external mechanical excitation [57]. (**c**) Typical rotary and sliding TENG architectures highlighting material configurations and electrode arrangements [58]. (**d**) Photographic and schematic examples of practical TENG devices illustrating transistor-based sliding mode [59].

TENGs operate in four fundamental working modes: contact–separation mode, lateral sliding mode, single-electrode mode, and freestanding triboelectric-layer mode [47]. Among these, the contact–separation mode is the most widely adopted, where two triboelectric layers repeatedly contact and separate under external mechanical excitation to generate alternating electrical output. This mode offers high performance, simple fabrication, and compatibility with flexible and compressible biowaste-based materials [48]. The lateral sliding mode relies on in-plane relative motion between triboelectric surfaces, causing charge redistribution through changes in the effective contact area, and is particularly suited for rotational or shear-type mechanical inputs. The single-electrode mode simplifies device architecture and facilitates integration into wearable electronics and self-powered sensors, although it generally delivers lower output due to charge dissipation to the environment [49]. In contrast, the freestanding triboelectric-layer mode employs a mobile triboelectric layer moving between two fixed electrodes, reducing charge-screening effects and enabling high energy conversion efficiency, especially in large-area and rotary systems [50]. The fundamental mechanisms and device configurations are schematically summarized in Figure 2. As shown in Figure 2a, triboelectric charge generation originates from interfacial contact electrification between materials with different electron affinities, followed by electrostatic induction that drives electron flow through an external circuit during mechanical motion [51]. The electron cloud–potential well model provides a theoretical framework for understanding atomic-scale electron transfer, while the persistence of surface-bound charges after separation enables sustained energy conversion. Figure 2b illustrates the dynamic evolution of charge distribution and electrical output under cyclic mechanical stimulation, emphasizing the relationship between surface charge density and electrode potential difference. Representative device architectures, including rotary and sliding TENGs, depicted in Figure 2c,d, demonstrate practical implementations of these principles. These schematics highlight the critical roles of surface chemistry, contact geometry, and motion mode in governing TENG performance—factors that are especially significant for biowaste-based triboelectric materials, whose surface functional groups, hierarchical structures, and mechanical compliance strongly influence charge-transfer behavior.

Open-circuit voltage (Voc), short-circuit current (Isc), and transferred charge (Q) are the most commonly used parameters to evaluate the performance of TENGs and power density [52,53]. Of these parameters, transferred charge is, in many cases, considered to be the most intrinsic measure of triboelectric performance since it directly reflects surface charge density [54]. Power density, measured either by area or volume, allows for comparison of the power output of different device sizes and configurations. Cyclic stability, mechanical durability, and environmental stability, such as resistance to humidity and temperature fluctuations, are also vital for practical deployment [55]. In the case of biowaste-based TENGs, biodegradability, biocompatibility, and life cycle sustainability should also be determined in addition to standard electrical quantities. To make a meaningful comparison between biowaste-based and conventional synthetic TENG systems, it is therefore necessary to establish uniform testing conditions and standardized reporting practices.

## 3. Sustainability Rationale for Biowaste-Derived Materials

Comparative life cycle energy analysis shows the sustainability is better in biowaste TENGs. Costs of processing biowaste materials are very low, at 0.1–1 kWh/kg (simple washing, drying, grinding, or mild alkali treatment at 60–80 °C), compared to 50–200 kWh/kg of synthetic fluoropolymers such as PTFE, which involves petrochemical synthesis and high-temperature extrusion. In 1–3 years of continuous operation, biowaste TENGs have a cumulative energy harvest of 10–500 kWh m^2^ (power density of 0.5–5 Wm of continuous mechanical power), which is more than 100–1000× the embodied processing energy. Even higher ratios are attained by carbonized biowaste hybrids (e.g., corn husk TENG-SC), since the hybrids can also be harvested and stored, and require days to weeks to payback against the years of silicon photovoltaics. Such a positive energy payoff, together with a negative material cost of waste avoidance, makes the biowaste TENGs an extremely sustainable alternative to more traditional energy-harvesting technologies.

The choice of triboelectric material plays an important role in defining the efficiency, stability, and scope of triboelectric nanogenerator (TENG) applications. In addition to electrical performance, material source, availability, processability, and environmental compatibility are critical design considerations for scalable energy-harvesting technologies [60]. Materials derived from biowaste combine inherent physicochemical strengths with sustainability advantages, providing a logical pathway toward energy-harvesting systems based on recycling natural resources in line with circular bioeconomy principles. Biowaste comprises continuous high-volume feedstocks, including agricultural wastes > 1.5 billion tons per year [61]: rice husks [62], wheat straw [63], sugarcane bagasse [64]; food and household wastes > 1.3 billion tons per year [65]: fruit peels [66], eggshells [67], coffee grounds [68]; and marine and animal by-products > 40 million tons per year [69]: fish scales [70], shells [42]. In contrast to engineered synthetic polymers that require petrochemical resources and complex supply chains, biowaste is available on the global market without the need for further extraction or energy-intensive production steps [71]. Completely biowaste-derived or minimally processed biowaste materials save 50–80% of the embedded carbon footprint and manufacturing costs associated with conventional PTFE or PDMS production, while simultaneously addressing the accumulation of >650 million tons of polymer waste in landfills annually and enabling sustainable resource recovery in accordance with circular utilization concepts. Natural biomaterials possess abundant surface chemistry and hierarchical structures that are highly supportive of triboelectric energy harvesting. Biopolymers such as cellulose, chitin, collagen, and gelatin contain high densities of polar functional groups OH (5–8 wt%), –COOH (1–3 wt%), –NH_2_ (1–4 wt%), and –CONH_2_ (2–6 wt%), which enhance charge transfer via hydrogen bonding and dipole interactions, enabling high triboelectric polarity with minimal or no chemical modification. The effective contact area is enhanced by 2–10× through hierarchical microstructures (10–100 µm pores, 5–50 nm fibers), which, in turn, differentiate biowaste-based TENGs from rigid synthetic materials due to their exceptional mechanical compliance (Young’s modulus of 1–100 MPa). The ability to flexibly process materials (washing, drying, grinding: <0.1 kWh/kg energy) and further process them (carbonization at 400–700 °C, activation to achieve 1200–3600 m^2^/g surface area) allows for property customization without complicated synthetic chemistries, thereby facilitating distributed on-site production [39,72,73,74,75,76].

Biowaste-derived materials are inherently biocompatible, non-toxic, and biodegradable, broadening applicability in wearable electronics, biomedical sensing, and implantable devices while eliminating surgical removal requirements and reducing foreign-body reactions. Growing experimental evidence demonstrates that biowaste-derived TENGs achieve electrical outputs (150–400 V, 0.5–255 mW/m^2^ power density) comparable to or exceeding conventional synthetic systems. This performance parity arises from intrinsic material features’ surface chemistry richness, structural hierarchy, and mechanical adaptability rather than compromise, exemplifying convergence of functionality and environmental compatibility. When rationally selected and engineered, biowaste materials provide high-performance triboelectric energy harvesting while supporting scalable, responsible material utilization aligned with circular bioeconomy principles, simultaneously addressing global waste management challenges and enabling sustainable, self-powered electronics for 21st-century distributed autonomous systems [71,73,74,75].

## 4. Life Cycle Assessment and Circular Economy Integration

Life Cycle Assessment (LCA) of biowaste TENGs finds that negative material cost (waste avoidance), low processing energy (0.1–1 kWh/kg vs. 50–200 kWh/kg in PTFE), and emission avoidance in landfill/incineration lead to carbon footprint reductions of 70–90% over synthetic polymer alternatives. Cradle-to-grave analysis (ISO 14040/44) [77] reports global warming potential savings of 210 kg CO_2_-eq/m^2^ TENG over 2 years; aggregate electrode metals (Al, Cu) can be eliminated through thin-film designs and recycling and can save 2–10 kg CO_2_-eq/m^2^ TENG through displacement of grid electricity (0.4–0.6 kg CO_2_/kWh) and chemical fertilizers (compost credits). These are concepts of the circular economy, which implement closed-loop material flows: biowaste sourcing avoids 1.5–1.3 billion tons/year agricultural/food waste releases, modular designs allow for the recovery of end-of-life electrodes (>95% Cu recyclability), and biodegradable tribolayers exclude microplastic risks [78].

Technology transfer advancements span prototypes to commercialization across fields: textile manufacturing scales electrospun cellulose TENG yarns via roll-to-roll processing (1–10 m/min, pilot lines in South Korea), IoT agriculture deploys corn husk TENG sensor networks (100-unit field trials powering DHT11/LoRa nodes, India 2024), wearables integrate fish scale biopolymer patches (Samsung/KAIST collaboration, skin-conformable ECG monitoring), and building energy harvesting validates 1 m^2^ rooftop panels (EU Horizon projects, 50 Wm wind-driven output). Licensing agreements with waste processors (coffee grounds to SCG carbon electrodes) and standardization efforts (IEC TENG task force) accelerate market adoption, projecting 10–100 MW annual deployment by 2030 for distributed IoT power [79].

## 5. Biowaste-Derived Triboelectric Materials

Scalability roadmap of biowaste TENGs involves moving the 3 × 3 cm prototypes used in the laboratory to the industrial textile and panel application and maintenance of the mechanical durability of the biopolymer used in continuous load. Phase 1 (short term): Roll-to-roll coating and electrospinning of biowaste inks (corn husk powder, cellulose nanofibers) onto continuous textile substrates (cotton, nylon) at 1–10 m/min rates; fabric-scale TENGs (1 m^2^) were obtained through embroidery or screen printing of segmented electrode patterns. Phase 2 (mid-term): textile weaving/knitting with core–shell yarns (biopolymer core, carbon electrode sheath) of conformable garments with retention of >80% of output after 10,000 stretch cycles (30 strain) can be achieved. Phase 3 (industrial): modular assembly of panel (10 m^2^ to 100 m^2^) with plasma-etched biowaste films pressed between carriers made of PET to harvest energy on the rooftop/building. Crosslinking (glutaraldehyde with chitosan/gelatin), nanocomposite reinforcement (25–50 wt% of cellulose nanocrystals), and multilayer encapsulation are used to improve biopolymer stability to withstand greater than 90% performance stability after 1 million cycles of continuous loading with 5–50 N, and are comparable to synthetic performance. Textile TENG pilot demonstrations indicate that textile TENGs can power 6–12 months of IoT wearables with 95% mechanical retention.

Biowaste TENGs can be biodegraded through enzymatic hydrolysis of biopolymer bonds in the soil, which has been demonstrated by ISO 14855 [80] composting respirometry (CO_2_ evolution), ASTM D5338 mass loss testing, and SEM erosion analysis, and electrical characterization in situ with impedance spectroscopy revealed 5–20% charge retention after biodegradation. The electrical characterization comes with open-circuit voltage (V_oc_) and short-circuit current (I_sc_) under different linear frequencies. Reproducibility exploits thermoplastic recycling of gelatin/chitosan (hot water/acetic acid redissolution) and cellulose (NaOH recovery, 80% performance after 23 cycles), and carbonized electrodes allow for modular tribolayer replacement, with 90% biodegrading in 6 months, providing the capability of a true circular economy as validated by accelerated aging and field experiments.

### 5.1. Coffee Ground and Bio-Polymer Systems

**Figure 3:**
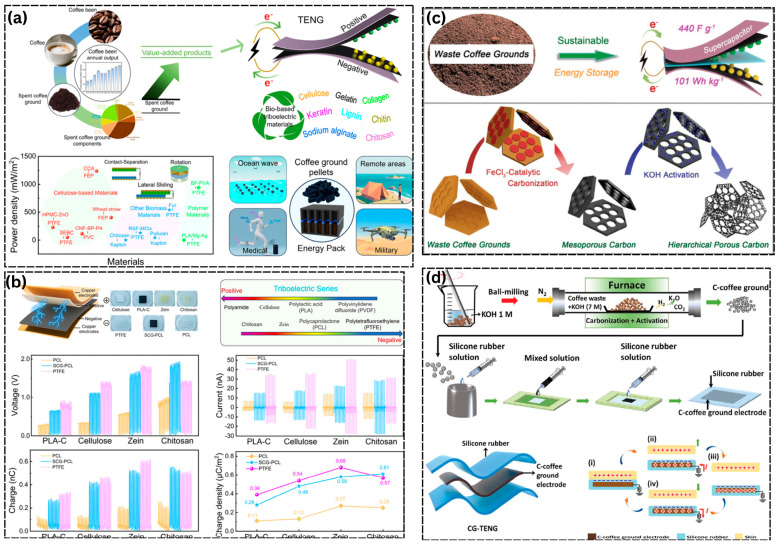
Triboelectric materials derived from biowaste of used coffee beans and biopolymer systems. (**a**) Schematic representation of spent coffee grounds as a value-added biowaste resource for triboelectric nanogenerators (TENGs), including material composition, triboelectric performance, and application scenarios. (**b**) Comparative triboelectric output properties of biopolymer-based materials, showing trends in voltage, current, and charge density compared with conventional triboelectric materials and their positions in the triboelectric series [88]. (**c**) Conversion of waste coffee grounds into functional carbon materials through catalytic carbonization and activation processes, resulting in mesoporous and hierarchically porous carbon structures for energy harvesting and storage. (**d**) Construction pathways and working principles of coffee ground-based TENG sensors, demonstrating their functionality as triboelectric layers, electrodes, and flexible device systems [96].

The growing coffee industry across the globe generates massive amounts of spent coffee grounds, which represent a simple and low-cost biowaste stream rich in carbon. These residues have traditionally been considered a disposal burden, but they have now emerged as useful functional materials for TENGs [81]. Figure 3a demonstrates that spent coffee grounds can be systematically upgraded into active triboelectric components through direct utilization by forming composites with biopolymers or by thermochemical transformation into conductive carbon structures. The inherent structure of coffee waste, consisting of lignocellulosic fibers, polysaccharides, proteins, residual oils, and aromatic carbon sources, provides a chemically rich and structurally diverse platform for trapping and storing triboelectric charges. Coffee-ground-derived materials are particularly promising for triboelectric applications because they exhibit desirable rough microtextured surfaces and contain a high density of polar functional groups when used either as direct friction layers or as fillers in polymer matrices [82,83,84]. Table 1 contains recent studies regarding coffee- and biopolymer-driven TENGs. These properties facilitate effective interfacial contact and charge transfer under mechanical stimulation. Moreover, coffee-based phases can be readily integrated with a wide range of bio-based polymers, including cellulose, gelatin, collagen, keratin [85], lignin, chitin, sodium alginate, and chitosan, enabling fully or semi-biodegradable TENG architectures. The rationale behind such hybrid systems is that they combine the mechanical flexibility of biopolymers with the high charge density of coffee-derived components, allowing the device to be optimally tailored for diverse application conditions [86,87,88].

**Table 1:** Coffee ground- and biopolymer-based BW-TENG materials: key parameters and performance metrics.

Material/System	Biowaste Source	Device Configuration	Electrical Performance	Key Advantages	Environmental Stability	Applications	Refs.
Spent coffee ground (SCG) composite TENG	SCG collected from cafés, dried (60 °C, 12 h)	SCG-PCL film (tribopositive)/PTFE film (tribonegative), vertical contact–separation mode	V_oc_ = 80–120 V; I_sc_ = 2–5 µA; charge density 67–127% of PTFE; Pmax = 0.5–2.5 W/m^2^	Low-cost abundant feedstock (>8 million tons/year globally)	Exhibits low sensitivity to humidity: even at 90% RH	Self-powered switches, portable energy packs for remote areas	[89]
Carbonized SCG electrodes (activated carbon)	SCG pyrolyzed (400–700 °C, N_2_ atmosphere, 2 h)	Integrated as a conductive electrode in hybrid TENG supercapacitor systems	BET surface area 1200–3600 m^2^/g; specific capacitance 131–280 F/g; electrical conductivity 10–50 S/m	Dual functionality: electrode + energy storage	-	Self-charging power units for IoT sensors	[90]
Cellulose (from coffee filters/agricultural residues)	Coffee filters or lignocellulosic biomass treated with NaOH (3–10 wt%, 80 °C, 2 h)	Cellulose film (tribopositive)/PTFE or FEP (tribonegative), contact–separation mode, Al or Cu electrodes	V_oc_ = 50–200 V; I_sc_ = 1–10 µA; Pmax = 10–300 mW/m^2^; crystallinity index 48–65%	Abundant global availability; simple alkali treatment	Maintaining ~70–85% output retention at 60–80% RH for operational lifespans exceeding 10,000 cycles	Ocean wave energy harvesting	[91]
Chitosan (crustacean shell biopolymer)	Chitosan (deacetylated chitin from crab/shrimp shells) dissolved in acetic acid (1–2 wt%)	Chitosan film (tribopositive)/PTFE or nylon (tribonegative), contact–separation or single-electrode mode	V_oc_ = 30–100 V; I_sc_ = 0.5–5 µA; Pmax = 5–50 mW/m^2^; amino content 1–4 wt%; degree of deacetylation 75–95%	Excellent biocompatibility (FDA-approved); antibacterial properties	Retains 94.5% output after 20,000 cycles at humidity (40–60% RH); stable over 1000 h	Implantable bioelectronics, wound-healing patches	[92]
Gelatin/collagen (animal protein biopolymer)	Gelatin (hydrolyzed collagen from animal skin/bones) dissolved in water (5–15 wt%)	Gelatin film (tribopositive)/PDMS or PTFE (tribonegative), contact–separation mode	V_oc_ = 20–80 V; I_sc_ = 0.3–3 µA; stretchability > 100%; biodegradation time 2–4 weeks (PBS, 37 °C); amide content 2–6 wt%	Transient/biodegradable electronics; excellent tissue compatibility	Retains 85% output after 60 days at ambient humidity	Biodegradable implants	[93]
Sodium alginate (seaweed polysaccharide)	Sodium alginate (from brown seaweed) dissolved in water (2–5 wt%)	Alginate hydrogel (tribopositive)/silicone or acrylic elastomer (tribonegative), contact–separation or single electrode	V_oc_ = 110–300 V; I_sc_ = 14.6–46.3 µA; 15% RH to 95%RH	Self-healing capability through dynamic ionic crosslinks	Retains stable output performance after >10,000 cycles under 50% RH	Transparent wearable electronics, robotics	[94]
Keratin (wool, hair, feather protein)	Keratin extracted from wool/hair/feathers via sulfitolysis or steam explosion	Keratin film (tribopositive)/nylon or PTFE (tribonegative), contact–separation or lateral sliding	V_oc_ = 25–500 V; I_sc_ = 0.4–4 µA; cysteine content 7–15%; disulfide crosslinks enable self-healing	Abundant textile/poultry industry waste (>15 million tons/year)	Stable > 10,000 cycles at ambient RH (40–70%)	Textile-integrated wearables	[95]

Figure 3b also demonstrates that triboelectric materials derived from biowaste are not necessarily inferior to traditional fluorine-containing triboelectric polymers [97]. In material performance comparisons of silk fibroin biopolymer from silkworm waste with MXene (Ti_2_C_2_T_x_) [98], cellulose- and lignocellulose-based triboelectric layers such as paper, wheat straw, and other biomass-derived sheets operating in contact–separation, lateral-sliding, or rotational modes routinely achieve power densities in the range of 10^2^–10^3^ mW m^−2^. These values approach, and in some cases rival, those of benchmark synthetic polymers such as PTFE and FEP under comparable testing conditions. Coffee ground-based composites can also operate within this high-performance regime when paired with appropriate counter-surface and electrode designs, indicating that with careful control of surface chemistry, microstructure, and device architecture, the inherent variability of biowaste feedstocks can be effectively mitigated [99]. Beyond direct triboelectric applications, spent coffee grounds can be catalytically carbonized and chemically activated to produce mesoporous or hierarchically porous carbons with large surface areas and high electrical conductivity [81,88,89,90]. When incorporated into BW-TENGs, these carbonized coffee-derived materials can function as flexible electrodes or charge storage layers, as schematically illustrated in Figure 3c, thereby enhancing output stability and device lifetime. Such porous carbon structures further enable multifunctional device concepts, including hybrid energy-harvesting and energy storage systems. To optimally utilize the energy of the TENG harvesters with the biowaste-based supercapacitor electrodes, electrical impedance matching should be done attentively. TENGs produce high-voltage and low-current pulses with high internal impedance, and carbon electrodes made of coffee grounds or corn husks have low resistance but high capacitive reactance. The high coupling requires that the optimal load resistance of the TENG match the impedance of the supercapacitor at the operating frequency, which is done by designing the electrode strategically, by the choice of an electrolyte and by use of intermediate power management networks such as bridge rectifiers and voltage regulators. Most of the energy produced is lost when not matched properly, and hybrid systems are much more efficient in storage, with an optimized interfacing, allowing self-powered IoT devices to become practically deployable, where they use entirely biowaste-based materials, for example, combining triboelectric generation with supercapacitive charge storage [68,89]. Meanwhile, Figure 3d shows the coffee ground-based TENG sensors, demonstrating their functionality as triboelectric layers, electrodes, and flexible device systems using silicone rubber.

In this context, three auxiliary functions of coffee waste can be implemented in biowaste-based TENGs. First, it serves as a tribopositive or tribonegative friction material with a heterogeneous organic structure and intrinsic surface roughness, which promotes efficient charge generation. Second, after thermochemical transformation, coffee-derived porous carbon acts as a mechanically stable and electrically conductive electrode or interfacial layer that enhances charge collection and storage [100]. Third, coffee-based materials in pelletized or composite form enable modular and scalable device concepts, including portable energy packs for use in off-grid or remote settings. Coffee ground systems exemplify how a single, high-volume biowaste stream can form the basis of a general and scalable material platform for harvesting triboelectric energy in environmental, wearable, medical, and IoT applications when combined with other bio-based polymers positioned across the triboelectric series [101].

### 5.2. Fruit Peel- and Eggshell-Derived Triboelectric Materials

**Figure 4:**
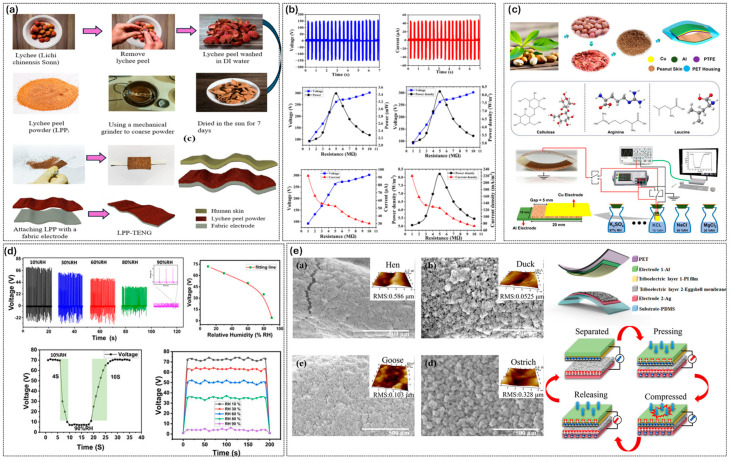
Fabrication and performance of food waste-based triboelectric nanogenerators (TENGs). (**a**) Fabrication process of lychee peel powder–based TENGs (LPP-TENGs). (**b**) Integration of LPP-TENGs with flexible textile electrodes and their electrical output characteristics, including voltage, current, power density, and humidity dependence [101]. (**c**) TENG architecture and molecular-level contributions to charge transfer in peanut skin powder-based sensors, along with self-powered sensor operation under various ionic conditions. (**d**) Electrical and environmental stability of food waste-derived TENGs under cyclic operation and across different humidity conditions [102]. (**e**) Surface morphology of eggshell membranes from various species and their relationship to triboelectric performance, including the operating mechanism of eggshell membrane-based TENGs, illustrating contact–separation-induced charge generation and device functionality [67].

Besides coffee ground systems, other relevant food waste sources, such as fruit peels and eggshell membranes, can also be used as triboelectric materials, as systematically summarized in Figure 4. The fruit peel category exploits the lignocellulosic biomass structure and high pectin content of agricultural byproducts, while eggshell membranes derive their functionality from a naturally fibrous collagen network. Despite their large availability and low cost, significant differences in microstructure, chemical composition, and surface morphology between these two classes dictate distinct triboelectric positioning and performance behavior. Consequently, careful material selection and processing optimization for specific applications are crucial.

In Figure 4a, LPP (lychee peel powder) TENGs are prepared using a very simple, minimal processing approach in which lychee peels are washed, sun-dried, ground into a coarse powder, and then deposited onto stretchable fabric electrodes without subjecting the peels to complicated chemical manipulations. The resulting lychee-based tribolayer is skin conformable and mechanically compliant, allowing it to interface with human tissue or textile counter layers under bending and pressing forces. Load-resistance sweeps indicate that optimized LPP TENGs can provide open-circuit voltages of 150–300 V and instantaneous power outputs sufficient to drive LED arrays and low-power microcontrollers. Notably, humidity-dependent measurements (10–90% RH) demonstrate that, despite a reduction in output voltage at high humidity due to surface water adsorption, a phenomenon characteristic of all hygroscopic biopolymer TENGs, the LPP TENG retains stable and repeatable signal generation across a wide humidity range. Inherent performance loss of 30–40% in the humidity of biowaste TENGs is caused by adsorption of moisture on hygroscopic surfaces and thereby blocks triboelectric charge and facilitation of ion-driven leakage currents. Technical mitigation methods include hydrophobic encapsulation with thin PDMS or parylene-C coats (50–200 nm thick) that preserve gas permeability and block ingress of liquid water while allowing evaporation, superhydrophobic surface engineering through plasma etching, laser ablation or fluorsilane grafting to form water contact angles over 150° and minimize adsorbed layers of water, and fibrous architecture (e.g., electrospun cellulose/chitosan nanofiber matrices). Other techniques are based on the use of charge excitation via corona poling or UV ozone modification to form persistent surface dipoles that are not sensitive to humidity screening, or the use of water-assisted enhancement in ion gels such as PVA/LiCl, where controlled hygroscopicity is a way to enhance tribopositive. These methods regularly recover 70–90% of dry performance at 40–90% RH, which allows biowaste TENGs to operate reliably in wearable and outdoor IoT deployments, supporting their suitability for self-powered sensing applications under varying environmental conditions, as shown in Figure 4b. The performance of lychee peel systems is representative of general trends observed in citrus and melon TENGs. Devices based on pomelo peel derivatives with naturally porous microstructures can reach high power densities of approximately 255 mW/m^2^ and can function as dual-mode energy harvesters and humidity sensors, whereas watermelon rind Citrullus lanatus TENGs composed of cellulose and pectin matrices exhibit comparable performance and can operate continuously for up to three months. Compositional analysis shows that citrus and melon peels contain 13–24% pectin, with a high proportion of methoxyl pectin (63–72% degree of esterification), which serves both as a source of mechanical cohesion and as a charge donor through hydroxyl and carboxyl functional groups, thereby enabling effective triboelectric performance. Collectively, these findings highlight that fruit peel-based TENGs represent a highly versatile material platform, with structurally diverse properties whose behavior can be tuned through the selection of cultivar and extraction conditions. Peanut skin powder (PSP) represents a mechanistically complementary pathway for valorizing nut-processing residues, distinct from fruit peels due to its elevated concentrations of proteins, amino acids, and lignocellulosic fibers.

In PSP-based TENG architectures, peanut skins are dried and crushed into powdered particles and then inserted between aluminum and PTFE/PET layers, forming a stacked triboelectric unit with a controlled air gap of 1.5 mm between the Cu/Al electrodes, which exploits both the cellulose scaffold and amino-acid-rich particles present in the skin matrix. This triboelectric hybrid structure imparts distinctive triboelectric properties: peanut-skin TENGs exhibit open-circuit voltages of 390–910 V (depending on the excitation mode applied) and instantaneous power outputs of 1.3–12 mW, which are significantly higher than those of most cellulose-based systems. Figure 4c shows that PSP, when used together with optimized spacing between Cu/Al electrodes and engineered air gaps, can be integrated into compact devices whose output is sensitive to normal force and ambient relative humidity, and that force–voltage linearity (R^2^ > 0.89) and power-delivery stability can be achieved when excited at a frequency of 10–20 Hz. A PSP SPHS exhibits an open-circuit voltage of ≈162 V and an instantaneous power of ≈2.2 mW at 20 MΩ when a PSP TENG is integrated with a resistive PSP thin film, and it can power 150 LEDs and electronic calculators without external power, as shown in Figure 4d. Similar lignocellulosic nut wastes, such as almond, walnut, and pistachio shells, have been compared and are found to routinely produce power densities of 10^2^–10^3^ mW/m^2^, placing nut-based TENGs among the highest-performing plant-based triboelectric materials reported so far and listed in Table 2. This performance difference between nut-waste-based TENGs and simple cellulose films is most likely due to the synergistic contribution of protein-derived functional groups and the inherently higher porosity of nutshell microstructures; however, quantitative structure–function analyses of these contributions are still minimal [101,102].

Eggshell membranes (ESMs) also contribute to the collection of biowaste-derived triboelectric materials by exploiting collagen-rich fibrous networks that are fundamentally different from plant-derived cellulose. Figure 4e, lower right panel, compares scanning electron microscopy (SEM) and atomic force microscopy (AFM) images of hen, duck, goose, and ostrich ESMs and shows species-dependent variations in surface morphology and root mean square roughness, which directly modulate triboelectric output. These membranes are natural, thin (100–200 µm) proteinaceous layers found in avian eggshells and are incorporated into multilayer TENG devices using PET substrates, aluminum and Ag electrodes, and elastomeric backing layers. In operation, these membranes are used in the vertical contact–separation mode to exploit the intrinsic piezoelectric characteristics of type I, V, and X collagens. Methodical interspecies comparisons show that ostrich ESM exhibits the greatest surface roughness (RMS ≈ 0.328 µm), surface potential (≈9.5 kV), and dielectric constant (≈3.05), and therefore delivers open-circuit voltages of up to ≈300 V, short-circuit current densities of ≈0.6 µA/cm^2^, and maximum power outputs of ≈18 mW, approximately 1.1× to 4× higher than those of hen, duck, or goose ESM-based devices. The mechanistic origin of these performance variations is attributed to the higher pore density and specific surface area of ostrich ESM, which enhance the trapping and storage of electrons within micro- and nanoscale surface pockets, thereby increasing charge retention and output stability over repeated cycles. Notably, ESM-based TENGs exhibit excellent stretchability (>30% strain), biocompatibility with mammalian cell lines, and high output levels (sufficient to power 250 red LEDs, digital watches, and wearable biosensors), confirming their suitability for skin-mounted or implant-adjacent bioelectronic applications, where mechanical conformability and long-term tissue compatibility are critical. Nevertheless, several vital constraints remain: ESM performance degrades significantly at high humidity (>70% RH) and elevated temperatures (>50 °C), and the inherent variability of natural collagen structures makes it difficult to ensure batch-to-batch reproducibility, which is necessary for achieving commercial viability of ESM-based TENGs. Taken together, these observations demonstrate the remarkable structural and functional diversity available in food waste-derived triboelectric materials. Although all three material classes can deliver outputs comparable to those of synthetic polymers under laboratory conditions, real-world implementation still faces significant challenges, including material standardization, sensitivity to humidity, mechanical stability, and scalable processing, which remain key barriers to the broader commercialization of biowaste-based TENG technologies [67].

### 5.3. Marine Biowaste-Derived Triboelectric Materials: Fish Scales and Shells

**Figure 5:**
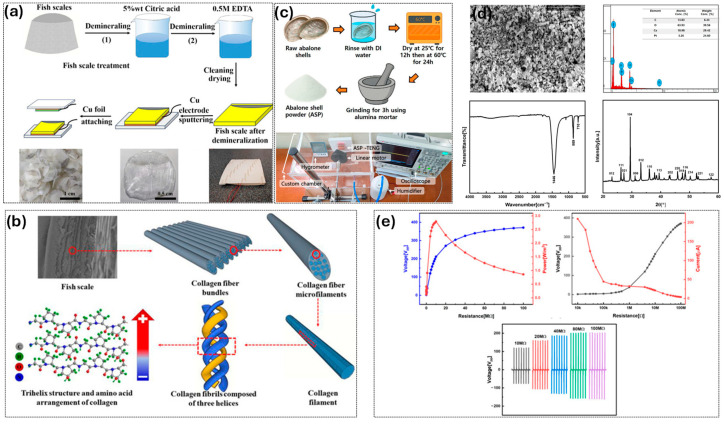
Triboelectric materials made of fish scales and shell biowaste. (**a**) The principle and process of fish scale-based triboelectric layer demineralization and device fabrication. Demineralization of triboelectric layers. Bases made of fish scales. Demineralization bases made of fish scales. Process: demineralization and device fabrication. This process involves the demineralization of triboelectric layers and the fabrication of triboelectric devices. (**b**) The hierarchical organization of fish scale collagen, between fiber bundles on the one hand and triple-helical filaments on the other hand, demonstrates the charge-active molecular organization [70]. (**c**) Processing of powder derived from abalone shells for use in triboelectric applications. (**d**) Morphological and chemical characterization of shell-derived materials on the surface. (**e**) Performance of fish scale-based and shell-based TENGs in terms of electrical output under cyclic mechanical excitation [42].

The marine processing industries also produce huge amounts of fish scales and mollusk shells, totaling more than 438 billion tons of shell waste per year, which can be upcycled into high-value triboelectric materials, as systematically shown in Figure 5. The structures and chemical characteristics of these marine biowastes are in marked contrast to lignocellulosic biomass because they contain collagen- and calcium carbonate (CaCO_3_)-based compositions, mineralized hierarchical microstructures, and intrinsic piezoelectric or triboelectric capabilities. These properties render biowastes of marine origin mechanically stable and versatile triboelectric systems for sustainable energy production. Fish-scale-based triboelectric nanogenerators (TENGs) are generally grown by a controlled demineralization process, as shown in the processing schematic in Figure 5a. Raw fish scales are treated sequentially with 5 wt% citric acid and then with 0.5 M EDTA to selectively remove inorganic calcium salts (16–59 wt%), leaving the underlying collagen structure intact. This partial demineralization directly modulates the molecular dipole gradient within the collagen matrix. At low mineral removal levels, residual CaCO_3_ phases mechanically constrain collagen fibrils and partially screen peptide bond dipoles, limiting intrinsic piezoelectricity despite preserved hierarchy. Sustainable electrospun graphene quantum dots from waste PET plastics also enable high-Q-factor TENG face masks for virucidal protection and self-powered theranostic wound healing [105]. In contrast, removal of approximately 16–59% of the mineral phase sufficiently releases lattice constraints and increases the effective volume fraction of oriented collagen triple helices, thereby enhancing spontaneous polarization (1–5 pC/N) without destroying the lamellar architecture or disrupting long-range dipole alignment. This milder treatment reduces collagen denaturation compared to aggressive HCl-based treatments, although at the expense of longer processing times [106]. The demineralized scales are washed, dried, bonded onto insulating substrates, and integrated with copper electrodes by sputtering or foil bonding to create flexible triboelectric films (approximately 100–200 μm thick) capable of functioning in contact–separation mode [107].

Figure 5b,d show the morphological characterization of both demineralized fish scales and raw abalone shells, where lamellar architectures and interconnected fibrous networks are retained. Preservation of type I collagen, characterized by typical amide bands and proline/hydroxyproline-containing triple helices stabilized by extensive hydrogen bonding, is confirmed by complementary FTIR, Raman, and XRD analyses. The hierarchical aggregation of amino acids into collagen is schematically illustrated in Figure 5b, where amino acids assemble into triple-helix fibrils (~300 nm), further organize into micron-scale fibers (1–5 μm), and align into lamellar layers across the scale. This hierarchical collagen arrangement produces permanent molecular dipoles associated with peptide bonds and hydroxyl side chains, leading to spontaneous polarization. Mechanical deformation disrupts dipole alignment, increasing charge separation and enhancing triboelectric output without external poling. Figure 5c shows the fabrication workflow and experimental setup, while electrical characterization of raw abalone shells under open-circuit conditions reveals voltages of 150–360 V and short-circuit currents of 10–400 μA, with stable operation over more than 10,000 cycles. These values are among the best reported for unmodified collagen-based triboelectric materials and highlight the applicability of fish-scale-derived TENGs for self-powered wearable sensors such as human motion monitoring (e.g., walking, finger tapping, and elbow bending). Growing research interest in marine-biowaste-based TENGs is reflected by the wide variety of systems summarized in Table 3.

Mollusk and abalone shells follow a complementary processing pathway that utilizes their CaCO_3_-rich composition and aragonite/calcite crystal hierarchies. Shells are washed and sequentially dried (Figure 5c) at 25 °C and 60 °C, followed by milling into abalone shell powder (ASP), with particle diameters ranging from sub micrometer to tens of micrometers. SEM–EDS and XRD analyses in Figure 5d show that abalone shells contain up to 77.6% aragonite, which is higher than that of typical oyster shells, imparting improved hardness, biocompatibility, and triboelectric charge affinity. ASP serves as an effective tribonegative filler when incorporated into polymer matrices (e.g., PDMS and polyurethane) or compressed into self-supporting pellets. Figure 5e further shows that voltage and power outputs are minimal at low load resistances (<10 MΩ), consistent with impedance-matching principles. The intrinsic piezoelectricity and permanent dipole polarization of fish scale collagen systems directly convert mechanical stimuli into electrical signals, whereas CaCO_3_-based shells operate predominantly through conventional triboelectric charge transfer driven by electron-affinity differences. This mechanistic distinction implies that hybrid collagen–CaCO_3_ systems can exhibit synergistic performance, as demonstrated in PVDF/fish scale-based systems showing more than twofold voltage enhancement compared to pure polymer films. Furthermore, both collagen and CaCO_3_ are biodegradable and biocompatible, enabling the fabrication of fully resorbable marine biowaste-based TENGs for transient bioelectronic applications. Despite these advantages, several challenges remain. Fish scale TENGs are sensitive to humidity and temperature, exhibit batch-to-batch variability due to biological heterogeneity, require time-consuming demineralization, and deliver lower voltages (7.4–39 V) compared to synthetic polymer TENGs. In addition, life cycle analyses are still limited, preventing accurate sustainability comparisons.

### 5.4. Medical and Pharmaceutical Solid Waste as Triboelectric Layers

**Figure 6:**
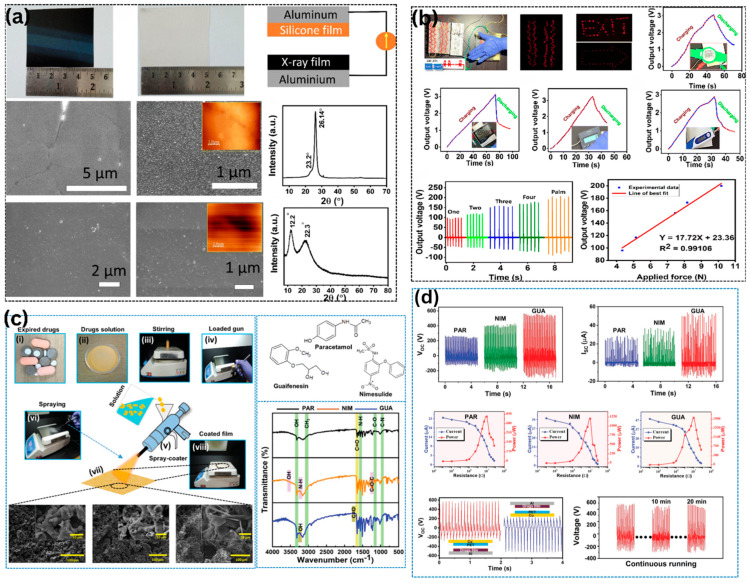
Triboelectric materials fabricated from biowaste and recycled waste for use in TENGs. (**a**) Structural composition and surface analysis of an X-ray film-based triboelectric layer, including optical images, SEM/AFM micrographs, and XRD patterns. (**b**) Electrical performance of the corresponding TENG under various mechanical stimuli, showing voltage generation, force dependence, and stable cyclic operation [113]. (**c**) Triboelectric surfaces derived from expired pharmaceutical substances fabricated by spray coating, along with their chemical structures, FTIR spectra, and surface morphologies. (**d**) Electrical characteristics of drug-based TENGs, including output voltage, current, power, and long-term operational stability under continuous mechanical excitation [114].

Other than food and marine biowastes, polymeric and pharmaceutical solid wastes, including discarded X-ray films, expired medicines, and clinical protective equipment, also constitute a significant waste stream that can be repurposed and directly used as triboelectric layers to harvest energy, as systematically shown in Figure 6. Medical plastic waste generated by the healthcare industry is estimated to be approximately 5.9 million tons per year worldwide, a figure that surged during the COVID-19 pandemic due to improper waste separation practices, resulting in more than 80% of medical waste being sent to landfills or incinerators regardless of contamination risks. At the same time, the disposal of expired pharmaceutical drugs, particularly analgesics such as paracetamol (acetaminophen), nimesulide, and guaifenesin, remains an ongoing environmental and public health concern. Paracetamol, for example, has been identified as accounting for 3.3–12.6% of total discarded tablet and capsule waste based on surveys in Danish hospitals, yet it is not fully removed during wastewater treatment (35–92% removal efficiency in sewage treatment plants), leading to ecotoxicological effects in aquatic environments. Together, these challenges motivate the development of biowaste-based TENG systems that can mitigate medical and pharmaceutical waste while simultaneously generating renewable energy [89,111,112].

TENGs based on X-ray films utilize the polyester/polyethylene terephthalate (PET) substrate and silver halide/gelatin photosensitive layers of discarded radiography films, as illustrated by the material photographs and SEM micrographs in Figure 6a. The fabrication pathway, schematically shown in Figure 6a, involves cleaning waste X-ray sheets with detergent and deionized water to remove surface contaminants, cutting the sheets into desired dimensions (3 cm × 3 cm), and bonding them to aluminum electrodes by heat pressing at 100 °C to ensure intimate contact and efficient charge transfer. Surface analysis using SEM and AFM (at 5 µm and 1 µm scales, Figure 6a, together with XRD patterns, reveals a semicrystalline polyester structure with clear diffraction peaks at 26.14° and 22.3°, along with relatively smooth and continuous polymer surfaces, except for a few embedded silver halide particles, confirming that polymer crystallinity is preserved after processing. When operated in a contact–separation configuration and paired with complementary silicone rubber or aluminum counter layers, X-ray film-based TENGs deliver stable AC output voltages of 110–150 V and peak power densities of 1.39 W/m^2^ under an applied force of 9 N and an excitation frequency of 3 Hz. Figure 6b presents time-resolved voltage traces that demonstrate high waveform repeatability with negligible signal drift during prolonged operation (>10,000 cycles), clear force-dependent voltage scaling, and the ability to power 420 commercial red LEDs, digital calculators, and wearable pedometers. These results clearly indicate the practical applicability of such devices in self-powered switches, touchpads, and low-power indicators for resource-constrained healthcare environments. Comparative studies of other medical polymer wastes, including saline bottles, polyvinyl chloride (PVC) gloves, and polystyrene packaging, further confirm that medical-grade plastics occupy favorable positions in the triboelectric series and can routinely achieve power densities of 1.46–8.78 W/m^2^, placing them on par with cellulose- and fruit peel-based TENGs derived from plant biomass.

Expired pharmaceutical drug TENGs represent another conceptually different waste valorization method that converts chemically complex pills into practical triboelectric layers. The fabrication workflow, shown in Figure 6c, is carried out in the following steps: first, expired pills (e.g., paracetamol, nimesulide, guaifenesin) are crushed into fine powders; then, they are dissolved or dispersed in appropriate solvents (ethanol, isopropanol, or water) under magnetic stirring to create solutions suitable for uniform coating; finally, the solutions are spray-coated onto conductive (aluminum) or insulating (glass, PET) substrates using commercial spray guns to form uniform thin films of 5–20 nm. The micro-/nanostructured morphologies, exhibiting interconnected porous networks, nanoscale grains, and phase-separated domains that increase the effective surface area and charge-generating capacity, can be observed under SEM imaging at different magnifications (Figure 6c, 50 µm scale bars). FTIR spectra in the wavenumber range of 4000–500 cm^−1^ confirm the presence of characteristic functional groups of each drug: paracetamol (amide I, C=O stretch, 1650 cm^−1^; amide II, N–H bending, 1560 cm^−1^; and phenolic O–H stretching, ~3300 cm^−1^), nimesulide (sulfonamide S=O stretching at 1340 and 1155 cm^−1^), and guaifenesin. Drug-film TENGs exhibit distinct voltage and current responses under periodic mechanical excitation (hand tapping, linear motors, or pneumatic actuators), which strongly correlate with their molecular structure and triboelectric positioning. A thorough electrical characterization of paracetamol (PAR), nimesulide (NIM), and guaifenesin (GUA), using time-resolved voltage and current traces, is presented in Figure 6d under controlled testing conditions (5 N force, 3 Hz frequency). Load-dependent power measurements in Figure 6d indicate well-defined power peaks at optimal load resistances of 20–40 MΩ, with maximum instantaneous power outputs of 163 mW (PAR), 145 mW (NIM), and 155 mW (GUA), which are sufficient to drive various LEDs, charge capacitors, and power microcontroller-based wireless sensors. Continuous operation for 10–20 min, as shown in Figure 6d, demonstrates good electrical stability, with less than 5% voltage attenuation, confirming the mechanical stability of spray-coated drug films under sustained cyclic loading. The mechanistic basis for the triboelectric differences among PAR, NIM, and GUA lies in their functional groups: the hydroxyl and amide groups of paracetamols, the sulfonyl and nitro groups of nimesulide, and the ether and hydroxyl groups of guaifenesin impart differing electron-donating and electron-withdrawing characteristics, respectively. These properties can be used to rationally select pharmaceutical compounds to tune device polarity and electrical response.

Critical comparative analysis demonstrates that medical and pharmaceutical waste-based TENGs present both advantages and persistent limitations, as reported in Table 4. On the positive side, X-ray films and pharmaceutical tablets are ubiquitous worldwide, produced across medical institutions, and require minimal preprocessing, primarily cleaning and spray coating, enabling simple scalability and distributed production. They are biocompatible (medical-grade plastics and FDA-approved active pharmaceutical ingredients), making them suitable for bioelectronic applications involving direct tissue contact. Moreover, synergistic value is achieved by combining waste elimination with energy production: every kilogram of X-ray film or expired pills diverted to TENG fabrication reduces landfill burden and associated greenhouse gas emissions from incineration while generating renewable electrical power. Nevertheless, several major challenges remain. First, pharmaceutical-drug-based TENGs exhibit high batch-to-batch variability because formulations differ across manufacturers and production lots (e.g., excipients, binders, coatings), complicating standardization and control. Second, the long-term environmental fate of spray-coated drug films is not well defined; although compounds such as paracetamol and nimesulide can biodegrade under certain conditions, their persistence in soils and potential leaching into groundwater necessitate rigorous life cycle environmental assessments before large-scale implementation. Third, X-ray film-based TENGs produce relatively low output voltages (110–150 V) compared with synthetic fluoropolymer standards (>500 V), requiring voltage-boosting circuits or hybridization with high-performance tribolayers to deliver practical output levels to typical electronic loads. Fourth, regulatory and health concerns surrounding the reuse of expired drugs even in non-ingestible applications pose barriers to adoption within conservative healthcare systems, underscoring the need for transparent communication and stakeholder engagement.

Overall, X-ray film and pharmaceutical waste systems confirm that medical industry waste streams are viable, high-volume feedstocks for triboelectric energy conversion, expanding the BW-TENG material portfolio beyond its current emphasis on agricultural and food wastes. However, translating these laboratory demonstrations into deployed waste-to-energy systems within healthcare facilities will require systematic resolution of material standardization, environmental safety, regulatory approval, and cost-competitiveness challenge issues common to all biowaste-derived TENG platforms.

### 5.5. Leaf- and Grass-Derived Triboelectric Materials

**Figure 7:**
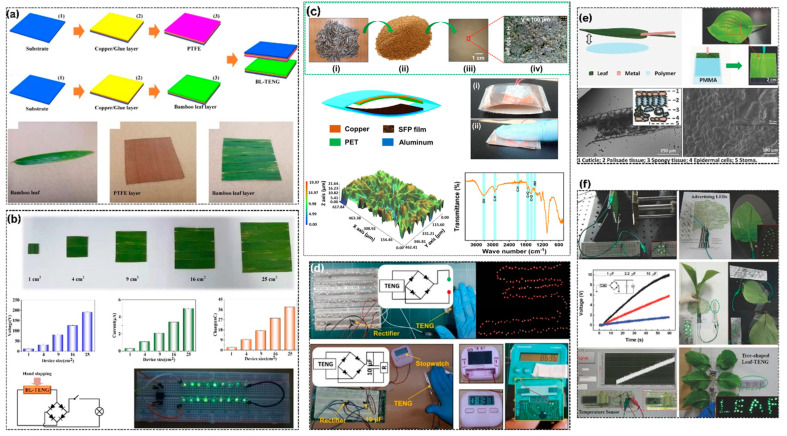
Triboelectric nanogenerators (BW-TENGs) based on leaf biomass and their demonstrations. (**a**) Schematic fabrication process of bamboo leaf-based TENGs, including layer assembly and comparison with standard polymer triboelectric layers. (**b**) Performance of bamboo leaf TENGs at different device areas, shown through optical images and area-dependent electrical output [32]. (**c**) Preparation of sunflower husk powder on triboelectric films, including surface morphology, thickness uniformity, and spectroscopic characterization. (**d**) Electrical performance of SFP-based TENG, demonstrating voltage output and LED lighting [117]. (**e**) Preparation of natural leaf materials on triboelectric films, including surface morphology, thickness uniformity, and spectroscopic characterization. (**f**) Applications of leaf-based TENGs in wearable electronics, sensing, and smart display systems [118].

Plant leaves and grasses form a valuable group of naturally organized, biodegradable triboelectric materials with unique hierarchical anatomical structures, low processing demands, and unparalleled mechanical compliance [116,117], as exemplified in Figure 7. Fallen leaves constitute a massive waste stream of 140–200 million tons per year in temperate and subtropical regions worldwide, which could be harnessed as high-volume feedstocks for triboelectric energy harvesting rather than being composted or burned. These leaves do not require energy-intensive chemical or mechanical processing, unlike extracted biopolymers (e.g., cellulose nanofibers, chitosan), and can be used with minimal pretreatment. As a result, they can be rapidly prototyped and readily scaled to support distributed energy-harvesting networks.

An example of a paradigmatic application of the direct lamination method is the bamboo leaf-based TENG (BL-TENG), as illustrated in the fabrication schematic in Figure 7a. Fresh bamboo leaves are washed with deionized water and then air- or oven-dried at moderate temperatures (60–80 °C for 6–12 h) to remove surface moisture without altering structural integrity or functional groups. The dried leaves are subsequently directly laminated onto a copper/glue-coated substrate, while a PTFE film placed on a counter copper electrode serves as the tribonegative counterpart. This lamination approach, which does not involve chemical extraction, in addition to biodegradation time in physiological media or nano-fibrillation, preserves the inherent microbridges, waxy cuticle layer, and mesophyll pore structure of living bamboo leaves, as confirmed by optical micrographs and SEM images in Figure 7a. These natural surface topographies significantly increase the effective contact area during contact–separation cycles, thereby enhancing charge generation and transfer. Scaling experiments over active areas ranging from 1 to 25 cm^2^ (Figure 7b) show that the open-circuit voltage (V_oc_), short-circuit current (I_sc_), and transferred charge (Q) scale approximately linearly with active area at a mechanical excitation frequency of 3 Hz, yielding V_oc_ ≈ 25 V, I_sc_ ≈ 1.20 µA, and Q ≈ 1.8 nC/cm^2^ of active area. A hand-slapped BL-TENG can easily drive a chain of commercial LEDs through a bridge rectifier, as shown in the photograph and circuit diagram in Figure 7b, clearly demonstrating its practical applicability for low-power green lighting and signaling applications. More importantly, BL-TENGs are highly robust: after 10,000 repetitive contact–separation cycles, only minimal attenuation of the output is observed, and the device is fully biodegradable in soil within 1–3 weeks, confirming effective end-of-life environmental management without the need for incineration or landfill disposal.

TENGs with hierarchical internal structures, such as intact broad-leaf TENGs, build upon this concept by exploiting the full anatomical design of living or freshly fallen leaves, as schematically depicted in Figure 7c,e. In this configuration, a healthy leaf is carefully cleaned and clamped between an aluminum or copper foil back electrode and a polymer support layer, typically a transparent polymer such as polyethylene terephthalate or polymethyl methacrylate (PMMA), to form a biological triboelectric composite. This structure incorporates the multiscale architecture of the leaf, including the macroscopic cuticle layer (1–5 µm thick), the photosynthetically active palisade and spongy mesophyll tissues, and the internal vascular network. The hierarchical three-dimensional system of leaf tissues, air gaps, and water-filled vascular cells enables a contact–separation cycling of a complex dielectric medium with a large effective permittivity and charge accumulation capacity that is significantly higher than that of flat cellulose films. The electrical measurements in Figure 7e indicate open-circuit voltages of 50–150 V and power densities of 1.8–5 W/m^2^ under open-circuit conditions using intact leaf TENGs across varying excitation frequencies, leaf species, leaf age, and moisture content, achieving performance comparable to or higher than that of processed cellulose-only TENGs. Remarkably, living leaves can be used without drying or chemical treatment, and even after leaf senescence (natural aging and drying), performance is not significantly affected for weeks to months, thereby increasing the feasible operational window for device deployment.

Silicone–grass fiber hybrid systems represent a hybrid pathway that combines processed plant fibers with elastomeric binders, as schematically shown in Figure 7c. Flexible triboelectric units are fabricated by mixing fibers with an adhesive (e.g., silicone rubber, PDMS, polyurethane), pressing the mixture into thin films (SFP, thickness = 0.1–0.5 mm), and placing them between copper, PET, and aluminum electrode layers. The resulting surface topography is finely textured due to randomly oriented fibrils, as confirmed by three-dimensional surface profiling (Figure 7c). The lignocellulosic composition (cellulose, hemicellulose, lignin) and the retention of carbohydrate functional groups (C–O, –OH) after hybrid fabrication are verified by FTIR spectra and X-ray analysis. Under contact–separation excitation (5–10 N force, 1–3 Hz frequency), silicone–grass fiber TENGs can generate open-circuit voltages of 80–180 V and short-circuit currents of 28–1 mA, corresponding to peak power densities of 2.1–6.3 W/m^2^ at optimal load resistances of approximately 20–50 MΩ. These values are competitive with those of pure plant fiber-based or synthetic polymer TENG systems. In addition, silicone rubber provides greater structural durability and ease of processing compared to fragile dried grass fibers while also enhancing mechanical compliance, enabling conformal contact with curved surfaces for wearable and integrated device designs.

Practical application examples demonstrate the feasibility of leaf-based TENGs for distributed energy harvesting and intelligent environmental sensing. Figure 7d,f illustrate the integration of BL-TENGs and intact leaf systems into large-scale visual and functional installations: tree-shaped TENG arrays composed of multiple leaf units wired in series and parallel operate LED strings at night to provide outdoor lighting and directional signaling; poster-like leaf assemblies drive temperature sensors, humidity monitors, and data loggers for automated agricultural and environmental measurements; and wearable leaf patches attached to textiles, backpacks, or hats harvest biomechanical energy from human motion (walking, limb flexing). Such demonstrations confirm that leaf- and grass-based TENGs can be readily implemented within existing infrastructure, ranging from urban parks and agricultural lands to personal wearable items, without the need for specialized installation or maintenance. This contrasts with traditional renewable energy systems (solar, wind), which typically require permanent infrastructure and professional maintenance.

An in-depth evaluation of weaknesses and research potential indicates that, although leaf- and grass-based TENGs offer outstanding sustainability and ease-of-implementation advantages, several factors dampen their near-term commercialization prospects. First, heterogeneity arising from differences in species, seasons, and growth conditions leads to performance variability: autumn leaves undergoing senescence exhibit 30–50% lower output than fresh spring leaves due to lignin accumulation and structural embrittlement. Second, moisture sensitivity remains a significant issue, with a 40–65% drop in output voltage at 80–90% relative humidity, as water molecules reduce the ability of triboelectric charges to accumulate on leaf surfaces; mitigating this effect would require protective encapsulation or hydrophobic coatings, which add cost and processing complexity. Third, the relatively short operational lifespan, ranging from days to weeks for fresh leaves and weeks to months for dried leaves before microbial degradation or structural disintegration, contrasts with synthetic polymer TENGs that can operate for many years, limiting applicability to scenarios that tolerate frequent component replacement. Fourth, current triboelectric performance (25–150 V, 1–8 W/m^2^) remains substantially lower than that of optimized synthetic polymer TENGs (>500 V, 50–200 W/m^2^), necessitating direct coupling with low-power microelectronics or cascaded voltage-amplification circuits to deliver usable power to conventional electronics. Finally, there are few comprehensive life cycle assessment studies of leaf-based TENGs quantifying environmental impacts such as carbon footprint, water usage, and end-of-life effects, making direct comparison with conventional renewable technologies difficult [119,120]. Taken together, leaf- and grass-based TENG systems demonstrate what can be achieved using minimally processed biowastes as sustainable triboelectric materials, in alignment with the principles of a circular bioeconomy. The capability to directly use fallen leaves without any chemical recovery, to incorporate devices into aesthetic and practical installations at community scales, and to produce results comparable to processed cellulose materials demonstrates that nature provides architecturally unique templates that promote mechanical strength and environmental sensing, which can be leveraged to engineer sustainable energy-harvesting systems [121,122]. However, these proof-of-concept demonstrations require systematic material engineering, encapsulation, and weather resistance to enable translation into commercially viable, long-lived, and continuously operating TENG systems, alongside comprehensive environmental life cycle assessment, in the face of persistent challenges common to all plant-derived TENG platforms.

## 6. Material-Processing and Surface-Engineering Strategies

**Figure 8:**
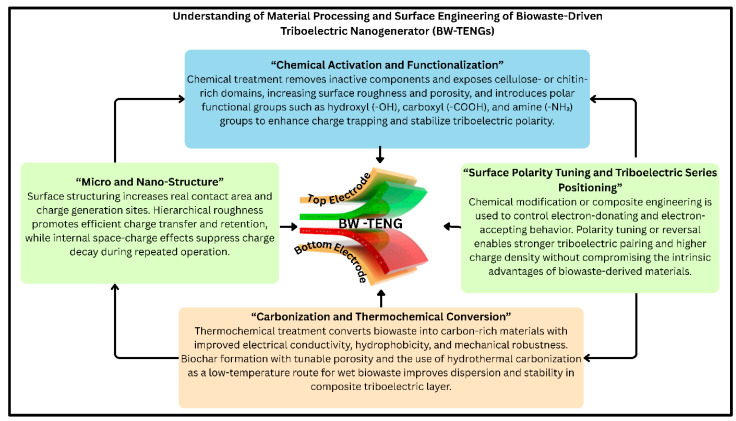
Schematic overview of material processing and surface engineering strategies for biowaste-driven triboelectric nanogenerators (BW-TENGs).

The successful use of biowaste in triboelectric nanogenerators (TENGs) requires regulated treatment to modify surface chemistry, morphological structures, and electrical characteristics, thereby bridging the gap between laboratory-scale demonstrators and commercially deployable energy-harvesting systems [91]. Chemical activation and functionalization are key strategies for purifying biowaste feedstocks and enriching surface functional groups involved in contact electrification [39,123]. Lignin–hemicellulose complexes can be cleaved by alkaline treatment with sodium hydroxide (NaOH, 0.5–10 wt%, 50–121 °C, 1–4 h), achieving 61–83 percent lignin removal, 29–66 percent cellulose recovery, and an increase in crystallinity index from 48 to 51, thereby exposing hydroxyl-rich cellulose surfaces that contribute to triboelectric charge-generation sites [124]. TEMPO-mediated oxidation preserves the cellulose backbone while selectively introducing carboxyl groups (0.8–1.8 mmol g^−1^) at the C6 position, resulting in open-circuit voltages of up to 152 V and charge-transfer efficiencies of 98 percent in TOCNF films; allicin-grafted TOCNF further enhances output by 2.6–15.2 times with charge-transfer yields of approximately 87% while also enabling nano-fibrillation with minimal energy input [125,126,127]. Sequential combined treatments, such as organogold pretreatment followed by NaOH processing, yield maximal cellulose recovery (≈66%) and delignification (≈83%) from sugarcane bagasse using reduced chemical inputs, underscoring that rational process integration is critical for industrial-scale viability. For that reason, surface engineering and chemical activation is required to strengthen its lifespan [128]. The key modifications are illustrated in Figure 8.

Thermochemical conversion transforms biowaste into carbon-rich materials with improved conductivity, hydrophobicity, and charge-storage capacity suitable for electrodes and hybrid TENG systems. Conventional pyrolysis (400–700 °C, inert atmosphere) progressively increases fixed carbon content (50–75%), decreases volatile matter (45–20%), and improves heating value (18–28 MJ/kg) through dehydration, decarboxylation, and carbonization reactions; surface area initially increases from 400–500 °C (50–200 m^2^/g BET), then declines above 700 °C due to pore collapse [129]. Hydrothermal carbonization (HTC) at subcritical water conditions (180–250 °C, 2–6 MPa, 0.5–8 h) enables wet biowaste conversion without energy-intensive drying [130], producing hydrochar [129] with increased hydrophobicity (water contact angle 90–120° versus < 30° for raw biomass) that mitigates humidity-dependent performance degradation. Chemical activation with KOH (3 M, 500–800 °C) generates hierarchical porous carbons with 1200–3600 m^2^/g BET surface area, 0.5–1.2 cm^3^/g micropore volume, and 131–280 F/g specific capacitance, suitable for supercapacitor electrodes and hybrid TENG charge storage layers; H_3_PO_4_ activation (400–600 °C) offers lower-temperature alternatives with tunable pore distributions and high surface functionality (–OH, –COOH) for ion adsorption and triboelectric charge trapping [131].

Triboelectric performance is boosted by micro/nano-structuring and surface polarity tuning by both geometrical and chemical processes. Photolithography and imprinting of 40–60 mm microstructures is optimum at 50 mm interstitial separation (87 W/m^2^ 255 times that of unpattern films), and laser etching produces higher voltages by 1.5–3 times contact area amplification and concentration of the electric field at edges [132]. Nanoscale roughness, carboxyl/carbonyl groups, and a 10-fold specific surface area increase are introduced with plasma etching (Ar, 50,100 W, 10 min or less) and do not cause excessive brittleness; materials are shifted towards the tribonegative end by plasma etching (CF 4 CF 8) 41 to 105 mV surface potential; and the 200 V voltage output is doubled (200 V vs. 100 V control) [133]. Internal space charge engineering through corona discharge (40,260 −2.0 −2.0 −2 charge density) doubles the charge retention by forming subsurface electric reservoirs, which produces open-circuit voltages of over 1000 V and power levels of over 10 W/m^2^. These four processing strategies together create a set of tools, which include chemical activation/functionalization, carbonization, micro/nano-structuring, and polarity tuning, combining to develop high-performance BW-TENGs with customized surface chemistry, morphology, and electrical properties that can be utilized in a variety of applications, including wearable electronics and distributed energy harvesting [133,134,135].

## 7. Applications of Biowaste-Derived TENGs

Triboelectric nanogenerators based on biowaste enable a wide range of applications in healthcare monitoring, environmental sensing, smart agriculture, and Internet of Things (IoT) networks, as outlined in Table 5. Unlike traditional energy harvesters that typically perform a single function, BW-TENGs can both capture ambient mechanical energy and detect environmental stimuli, allowing them to operate as integrated energy–sensing platforms. This dual functionality eliminates the burden of battery replacement and reduces the toxic accumulation of electronic waste. Such self-powered and autonomous sensing capability positions BW-TENGs as potentially transformative enabling technologies for distributed sensor networks, particularly in resource-constrained and grid-absent environments where battery replacement is economically prohibitive or logistically impractical.

### 7.1. Self-Powered Environmental and Chemical Sensing

BW TENGs are effective self-powered sensors for measuring chemical, physical, and environmental parameters through the direct utilization of stimulus-responsive triboelectric coupling. Humidity sensors make use of the hygroscopic behavior of cellulose-, pectin-, and protein-rich biopolymers, which show an exponential decrease in electrical resistance with increasing relative humidity, as water molecules adsorb onto polar functional groups (–OH, –COOH, and –NH_2_) and conduct protons through the adsorbed water layers [34,136,137]. A peanut skin powder (PSP) [104] self-powered humidity sensor (PSP SPHS) using a PSP TENG Table 5 demonstrated a sensitivity of 0.8 V/%RH and response/recovery times of 4/10 s, which are 2–5× faster than those of commercial resistive sensors, along with stable output over a 10–90% RH range for more than 30 continuous measurement days. The porous PSP thin film (thickness ≈ 50 µm), containing cellulose, hemicellulose, and lignin, is mechanically engineered to provide a large effective surface area (≈50 m^2^/g) and an interconnected pore structure, facilitating rapid moisture diffusion and reversible adsorption/desorption, thereby ensuring reproducibility. Likewise, an EASS cellulose/pectin self-powered humidity sensor exhibited a response time of 21 s, a recovery time of 14 s, and excellent long-term stability, further supporting the use of plant-derived polysaccharide TENGs as viable humidity sensors for agricultural greenhouses and indoor air-quality control systems [138].

Selective adsorption and chemi-resistive transduction in porous biopolymer composites are exploited in breath and volatile organic compound (VOC) sensing [139,140]. Polyethylene oxide–cuprous oxide (PEO/Cu_2_O) composite nanofiber TENGs can perform self-powered detection of ethanol with a response factor of 3.2 at a concentration of 200 ppm ethanol (90% RH) and can be applied in law enforcement for breath alcohol monitoring as well as in medical diagnostics, such as chronic obstructive pulmonary disease (COPD), by comparing exhaled acetone and other disease biomarkers [141]. The porous nanofiber structure (100–500 nm porosity) maximizes gas–solid contact area, while Cu_2_O nanoparticles provide catalytic sites for oxidative chemisorption, enhancing sensitivity and selectivity compared with pure polymer sensors [142]. High-spatial-resolution pressure and strain sensors (≈1 mm^2^ pixel size) enable tactile mapping for robotic grippers, prosthetics, and rehabilitation devices [143]. Fullerene carbon soot-doped polydimethylsiloxane composite film’s reliance on synthetic fullerene soot production via energy-intensive laser ablation limits its scalability and sustainability compared to direct biowaste-processing methods [144]. Chitosan–glycerol film TENGs are sensitive to gait patterns, foot-strike dynamics, and fall risk during walking by measuring spatiotemporal pressure distributions, exhibiting sensitivities exceeding 3.88 V/kPa and stable operation over more than 40,000 loading cycles. TENGs fabricated using hydrogels, with stretchability exceeding 200%, allow for conformable attachment to body joints (knee, elbow, wrist) for continuous mobility monitoring during physical therapy and athletic training, offering superior mechanical compliance and wearing comfort compared with rigid silicon-based strain gauges [145,146].

### 7.2. Wearable and Implantable Bioelectronics for Healthcare Monitoring

BW-TENGs have strong potential as wearable and implantable bioelectronics owing to their biocompatibility, mechanical compliance, and ability to harvest biomechanical energy from limb motion, arterial pulses, heartbeats, and respiration [147]. Wearable TENG-based cardiovascular monitoring systems can identify pulse waveforms, heart rate, and arrhythmias with sensitivity comparable to clinical photoplethysmography (PPG) sensors, without requiring external power sources or optical components [148]. Eggshell membrane-based TENGs, generating open-circuit voltages of 150–300 V under applied forces of 10–40 N, have enabled real-time pulse-wave data streaming to smartphones via integrated microcontrollers and Bluetooth wireless transmitters, allowing for continuous cardiovascular health monitoring for periods exceeding 30 days without battery replacement [149]. Woven textile-based TENGs integrated into clothing cuffs exhibit sensitivities of 3.88 V/kPa and can accurately record fine pulse-wave morphology, including the dicrotic notch, percussion wave, and tidal wave, providing clinically relevant information related to arteriosclerosis, hypertension, and heart valve diseases [148].

Multimodal TENG sensor networks integrating pulse, respiration rate, body temperature, and motion, as well as body sensor networks (BSNs) combining these physiological signals, enable personalized healthcare management, achieving energy conversion efficiencies greater than 57.9% when utilizing walking-induced inertial forces [150]. Transient and biodegradable TENGs eliminate the need for surgical removal by undergoing controlled degradation under physiological conditions. Chitosan/polyvinyl alcohol (PVA)-based TENGs fully degrade within 60 s in phosphate-buffered saline (PBS, pH 7.4, 37 °C), whereas poly (lactic-co-glycolic acid) (PLGA) and magnesium/polylactic acid (Mg/PLA) reed-film TENGs generate outputs of 0.176 V and 192 nA and maintain stable performance for up to 14 days prior to complete resorption, confirming their suitability for transient bioelectronic applications. Energy conversion efficiencies as high as 85% enable effective harvesting of low-frequency biomechanical energy from cardiac motion (~1 Hz), diaphragmatic contraction (~0.3 Hz), and gastrointestinal peristalsis (~0.05 Hz), sufficient to sustain intermittent sensing (1–5 measurements per minute) and wireless data transmission via Bluetooth Low Energy, where transmission packets typically last less than 100 ms. Furthermore, ultrasound-triggered transience enables on-demand device elimination: TENGs fabricated from poly(3-hydroxybutyrate-co-3-hydroxyvalerate)/PEG (PHBV/PEG) completely disintegrate under focused ultrasound (1 MHz, 2 W/cm^2^), thereby preventing foreign-body responses and reducing the risk of chronic inflammation [151].

### 7.3. Environmental and Marine Monitoring Systems

BW-TENGs are used in environmental monitoring systems that harness wind, rain, water flow, and ocean waves as power sources to operate remote sensors in grid-absent areas [152]. Wind- and water-flow-driven TENGs utilizing rabbit hair or fur can generate short-circuit currents of 14.8 mA, transferred charges of 130.9 nC, and peak power outputs of 3.54 mW at moderate wind speeds of 9 m/s, owing to the high charge-retention capacity of PTFE and continuous surface charge replenishment enabled by flexible rabbit-fur contact [153]. Such wind- and water-flow TENGs have been employed as power supplies for soil thermometers, humidity meters, LED indicator arrays, and wireless data loggers to enable real-time monitoring of environmental parameters such as temperature, humidity, water level, and salinity in offshore marine environments. Rotary TENGs with soft biomaterial contacts can deliver instantaneous currents of 15 µA and peak power outputs of 11.9 mW, supporting nighttime LED indicators, insect-capture electric grids for pest control, and continuous soil moisture and pH sensors that can operate for several months without replacement.

Multimodal energy harvesters capable of scavenging both wind and rain have been developed to enable year-round power generation under unpredictable meteorological conditions. In blue-energy applications, TENGs deployed at depths exceeding 100 m harvest ocean energy to power wireless sensor nodes via buoy-mounted or submerged contact–separation mechanisms, transmitting data on temperature, salinity, pH, and dissolved oxygen at intervals of 10–60 min over communication ranges of 2–10 km using low-power radio (LoRa), without batteries, for operation periods exceeding six months. In addition, cellulose fiber-based TENGs function as bifunctional particulate matter (PM) electrostatic filters and air-quality sensors, capturing PM2.5 and PM10 particles with efficiencies greater than 95% while simultaneously generating electrical signals proportional to airflow rate and particulate loading, thereby enabling self-powered air-pollution monitoring in urban and industrial environments [154,155].

### 7.4. Smart Agriculture and Precision Farming IoT Systems

Smart agricultural IoT systems can employ BW-TENGs to enable continuous environmental monitoring in grid-absent rural regions, supporting data-driven precision farming decisions. The high open-circuit voltage of 3.2 kV obtained with the corn husk composite pulsed TENG is attributed to its special pulsed TENG mechanism, which incorporates a large-area multilayer contact separation architecture with tribopositive, tribonegative, and high separation distances > 5 mm, which maximizes induced potential. The pulsed operation is facilitated by rotary movement caused by wind that produces sharp and high amplitude contact events, and by the hierarchical lignocellulosic microstructure of corn husk cellulose microfibrils 20–50 nm in diameter, nanoscale pores that increase charge trapping and transfer efficiency. This layout attains a transferred charge of 300 nC and stable output with higher wind speeds of 3–12 m/s, which makes it eligible for powering multichannel wireless sensing systems that monitor temperature (DHT11 sensor, ±2 °C), humidity (20–90% RH, ±5%), light intensity (BH1750 lux sensor, 1–65,535 lux), and soil moisture (capacitive sensor, 0–100% volumetric water content) at 15 min intervals, transmitting data over distances exceeding 1 km. Passive power management circuits incorporating bridge rectifiers, voltage regulators, and supercapacitor buffers achieve 54.5% energy storage efficiency, enabling continuous sensor operation under variable wind and rain conditions and autonomous circuit operation for more than 12 months without human intervention [156].

BW-TENGs can also provide electric-field stimulation to enhance agricultural productivity through bio-electrochemical growth promotion. The application of wind/rain hybrid TENG-generated electric field pulses (50–200 V/cm, 0.1–1 Hz, 30 min per day) to pea seedlings accelerates germination (26.3% reduction in time, from 7 to 5.2 days) and increases final biomass production (17.9%) compared with non-stimulated controls. These effects are attributed to electric field-induced redistribution of auxin and other nutrients, as well as reactive oxygen species (ROS), which activate stress-response and growth pathways. Cloud-based data analytics and real-time decision support for optimizing irrigation scheduling, early disease detection via anomalies in leaf temperature, and variable-rate pesticide application enabled by spatial pest pressure mapping are achieved through Bluetooth and LoRa connectivity [157].

### 7.5. Circular Economy: Waste Management and Energy Storage

Smart city waste management systems can utilize BW-TENGs within closed-loop circular economy models. Smart bins equipped with ultrasonic sensors detect real-time fill levels of color-coded biomedical receptacles (green: organic, blue: recyclables, yellow: plastics, red: hazardous) and achieve response times of approximately 3–16 min between automated alerts and the dispatch of collection vehicles [112]. TENG-powered IoT monitoring enables effective waste segregation, increasing recycling rates from approximately 35% (mixed-waste baseline) to about 72% (segregated collection) while reducing landfill burden. In this way, TENGs harvest energy to power monitoring systems for future waste streams, creating synergistic models in which present-day biowaste is used to energize the infrastructure required to manage future waste flows [158]. The practicality of BW-TENG power delivery to electronic loads has been demonstrated through energy-storage studies. Starch/BaTiO_3_ composite TENGs can charge 3.4 µF capacitors to 2.5 V within 90 s, enabling intermittent operation of high-power sensors (50 mW burst power, 10 ms duration). PDMS/multiwalled carbon nanotube (MWCNT) TENGs with full-wave bridge rectifiers can charge 1 µF and 3.3 µF capacitors to 1.1 V and 0.7 V, respectively, within 60 s, and subsequently power microcontroller units (Arduino Nano, ATmega328P; operating voltage 1.8–5 V, idle current 15 mA) and wireless transmission modules (nRF24L01; transmission power 11.3 mW) [12,159,160].

Direct LED powering further demonstrates the instantaneous output capability of BW-TENGs. Nopal cactus powder TENGs have been used to illuminate 116 ultra-bright LEDs (forward voltage 3.2 V, forward current 20 mA) and to operate digital calculators (operating voltage 1.5 V, operating current 0.1 mA). Onion skin-based TENGs can power 375 LEDs simultaneously (total power 0.8 mW/cm^2^, maximum power density 2.8 W/m^2^) for outdoor emergency lighting. Watermelon rind cellulose/pectin TENGs have sustained constant power delivery to 30 LEDs over three months without performance degradation, confirming long-term stability and environmental survivability. The highest-performing biowaste-based systems reported to date include rabbit-fur rotary TENGs, which generate open-circuit voltages of up to 10,000 V and peak power outputs of 1200 mW, sufficient to operate smartphones requiring 5 V and 1–2 A (5–10 W) input power, as well as low-power household appliances [12,34,112,159,160,161].

**Table 5:** Representative BW-TENG applications and performance metrics.

Application Category	BW Material	Device Configuration	Key Electrical Output	Demonstrated Functionality	Refs.
Self-powered humidity sensing	Peanut skin powder (PSP)	PSP film (tribopositiv)/PTFE (tribonegative), vertical contact–separation	V_oc_ = 162 V; P_max_ = 2.2 mW; sensitivity 0.8 V/%RH; response/recovery time 4/10 s	Self-powered humidity sensor (10–90%RH), powered 150 LEDs, digital calculator; stable operation > 30 days	[102]
	Ethanol acetate/sodium stearate (EASS) cellulose/pectin composite	EASS film/PTFE, vertical contact–separation	V_oc_ = 87 V; P_max_ = 98 mW/m^2^; sensitivity not specified; response/recovery time 21/14 s	Greenhouse humidity monitoring, agricultural IoT sensor node	[162]
Wearable healthcare monitoring	Eggshell membrane (hen, duck, goose, ostrich)	ESM (tribopositive)/PET or PDMS (tribonegative), wrist-mounted contact–separation	V_oc_ = 150–300 V; I_sc_ = 0.3–0.6 µA/cm^2^; P_max_ = 10–18 mW	Real-time pulse wave monitoring, heart rate detection (60–100 bpm), arrhythmia alerts; powered Bluetooth transmitter for >30 days	[67]
	Cellulose nanofibers (CNF) textile	CNF woven fabric/FEP film, chest-mounted contact–separation	V_oc_ = 30–50 V; I_sc_ = 3–8 µA; sensitivity 3.88 V/kPa	Respiratory rate monitoring (12–20 breaths/min), body-motion tracking; 57.9% energy-conversion efficiency from walking	[163]
	Magnesium/polylactic acid (Mg/PLA) reed film	Mg electrode/PLA film, contact–separation	V_oc_ = 0.176 V; I_sc_ = 192 nA	Biodegradable cardiac pacemaker (14-day operational lifespan before resorption); powered 22 LEDs	[164]
Environmental and marine monitoring	Rabbit hair/fur composite	Rabbit fur patches/PTFE film, rotary contact–separation (wind-driven)	I_sc_ = 14.8 mA; Q_sc_ = 130.9 nC; P_max_ = 3.54 mW (9 m/s wind speed)	Offshore marine sensor nodes (temperature, humidity, salinity); wireless transmission (LoRa, 2 km range); >7 weeks continuous operation (92% performance retention)	[165]
	Cellulose fiber filter	Cellulose nanofiber mat/PDMS or FEP, airflow-driven contact–separation	V_oc_ = 50–150 V; I_sc_ = 2–8 µA	Dual-function PM~2.5~/PM~10~ electrostatic filter (>95% capture efficiency) and air-quality sensor; self-powered airflow-rate monitoring	[166]
Smart agriculture IoT	Corn husk composite powder	Corn husk powder/PTFE film, vertical contact–separation pulsed TENG	V_oc_ = 3.2 kV; Q_sc_ = 300 nC	Multichannel wireless sensor (temperature ±2 °C, humidity 20–90%RH ±5%, light intensity 1–65,535 lux, soil moisture 0–100%); LoRa transmission 1.7 km range; 54.5% energy-storage efficiency	[154]
	Watermelon rind cellulose/pectin	Watermelon rind film/PTFE or Al electrode, contact–separation	V_oc_ = 150–200 V; P_max_ = 255 mW/m^2^	Powered 30 LEDs continuously for 3 months; greenhouse environmental monitoring; electric field crop stimulation (26.3% faster germination, 17.9% yield increase)	[34]
Energy storage and LED powering	Nopal cactus powder	Nopal powder/polyimide film, vertical contact–separation vibration-driven	V_oc_ = 14.56 V; P_max_ = 556.72 µW/m^2^	Illuminated 116 ultra-bright LEDs, powered digital calculator; charged 22 µF capacitor to 1.9 V in 900 s; stable operation > 27,000 cycles	[161]
	Onion skin	Onion skin layer/PTFE film, lateral-sliding contact	V_oc_ = 300–400 V; P_max_ = 2.8 W/m^2^ (0.8 mW/cm^2^)	Operated 375 LEDs simultaneously; emergency outdoor signaling; self-powered tactile sensor	[33]
	Carbon-coated paper wipes (C@PWs) from waste paper	Carbonized paper/polythene (plastic waste), contact–separation	V_oc_ = 245 V; I_sc_ = 3.5 µA; P_max_ = 0.61 mW (4 Hz, 25 N force)	Morse-code signal generator for autonomous emergency communication; charged 1 µF capacitor to 1.1 V in 60 s; powered 16 LEDs	[164]

**Figure 9:**
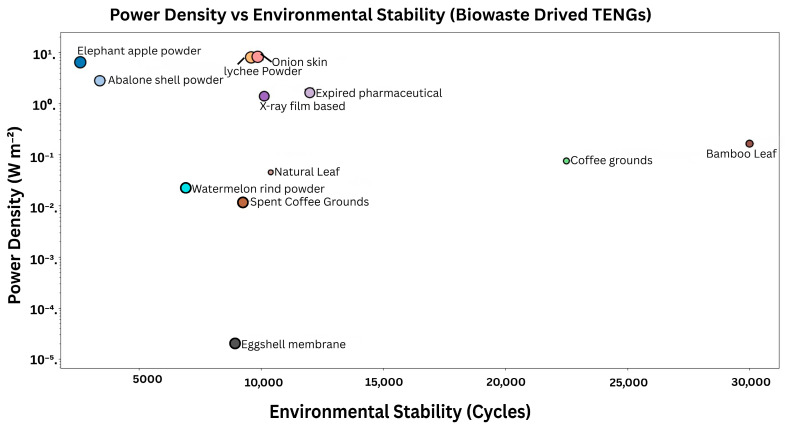
Power density vs. environmental stability for biowaste-driven TENGs. Data compiled materials including elephant apple powder [167], onion skin [33], lychee peel powder [101], abalone shell [42], expired pharmaceutical drugs [114], X-ray film waste [113], coffee grounds [96], spent coffee grounds [81], natural bamboo leaf [32], watermelon rind [34], and eggshell [67].

BW-TENGs are a constructive paradigm in energy-harvesting sustainability and autonomous electrification of sensor networks. Their triboelectric performance (voltages between 50 and 3200 V and power densities between 0.5 to 255 mW/m^2^), coupled with material biodegradability (full degradation in 60 s to 3 months, based on the material) and biocompatibility, have made it possible to build integrated architectures of devices where energy generation, sensing, and environmental remediation are convergent functions. The performance landscape in Figure 9 indicates that the highest performing (6.4 W m^−2^), with a greater stability of over 3000 cycles, is elephant apple powder (EA-TENG), and abalone shell, lychee peel powder, and bio-carbon systems (coffee grounds, spent leaves), which provide balanced trade-offs to various applications. The former can be defined as entirely sustainable, self-charged electronics that take advantage of the concept of the circular economy.

## 8. Challenges and Limitations in Biowaste-Based TENG Materials

**Figure 10:**
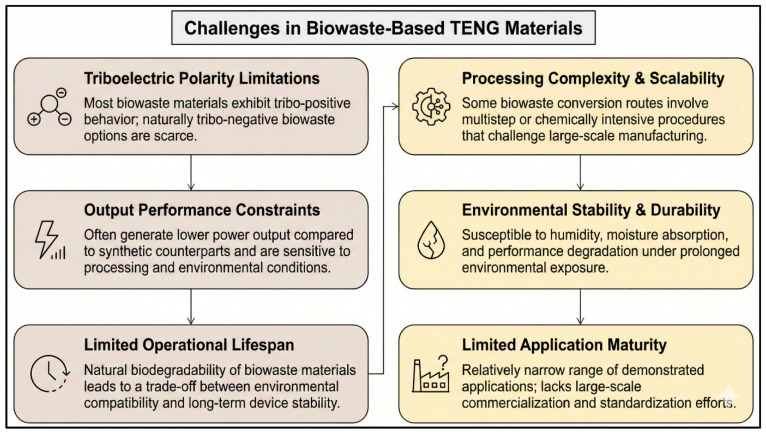
Triboelectric nanogenerators (BW-TENGs) based on biowastes are still facing challenges, such as the problem of polarity, processing and scalability, performance, environmental stability, short lifespans, and immature applications.

Inherent issues hampering the large-scale adoption of BW-TENGs, including variability in material composition, reproducibility, environmental stability, and performance limitations, are depicted in Figure 10. The tribopositive behavior of the majority of biowaste materials arises from cellulose-, protein-, or polysaccharide-rich structures, with fewer tribonegative choices and limited design freedom to form optimal triboelectric pairs. As a result, BW-TENGs typically produce lower power outputs (10–255 mW/m^2^, voltages 50–300 V) than optimized synthetic fluoropolymer TENGs (50–1200 W/m^2^, 500–10,000 V) due to lower electron affinity. A lack of consistency and reliability further constrains performance: seasonal variation, geographic provenance, cultivar differences, and storage conditions lead to variability in biowaste composition. For example, sugarcane bagasse exhibits cellulose contents of 8–15% [168,169] and lignin contents of 12–22%, with overall compositional variability of 15–27%, which directly translates into 20–35% output-voltage variation under standardized testing conditions [170]. Untreated flower-based TENGs [171] degrade after approximately 18,000 cycles due to finite particle-size distributions. Although chemical treatments such as NaOH extraction (0.5–10 wt%), TEMPO oxidation, and acid hydrolysis improve consistency (61–83% delignification and 29–66% cellulose recovery), these multi-step processes significantly increase fabrication costs (USD 1.50–8.00 per kg product), chemical consumption (5–20 L solvent per kg biomass), energy usage (2–8 MJ/kg), and processing time (4–48 h), thereby diminishing overall sustainability [172,173].

The absence of standardized collection, pretreatment, and fabrication protocols introduces substantial inter-laboratory variability in cellulose purity (55–83%), crystallinity index (48–65%), and triboelectric output (30–250 V), undermining scientific comparability and industrial practicality. Furthermore, the lack of international performance standards analogous to ISO 9806 [174] for solar collectors or IEC 61215 [175] for photovoltaic modules makes cross-comparison difficult, as widely varying testing conditions (e.g., electrometer impedance) are employed [74,176]. Current environmental sustainability and stability pose serious challenges to long-term deployment. Hygroscopic biowaste materials, particularly those with high concentrations of –OH, –COOH, and –NH groups, perform poorly under high-humidity conditions: adsorption of water molecules on polar sites reduces surface charge density through conductive leakage pathways, capillary condensation forms continuous water films that short the interface, and hygroscopic swelling alters surface morphology and reduces effective contact area [177]. As a result, textile-based cellulose TENGs can exhibit voltage drops of up to 52% (from 250 to 120 V). Temperature variations (from lows of 0 °C to highs of 150 °C) also adversely affect performance through thermal expansion stress, changes in dielectric constant, and accelerated charge loss. TENG output decreases by 40–70% at temperatures above 60 °C and by 15–35% near 0 °C due to polymer softening, increased charge recombination, and reduced charge retention; operation below 0 °C further causes embrittlement and mechanical failure unless protective measures, such as hydrophobic encapsulation, are applied [178]. Mechanical degradation further limits active lifetime: untreated plant-based TENGs typically fail after 18,000–50,000 cycles due to surface abrasion (5–20 µm wear depth), interfacial delamination, and microcrack propagation. This lifetime is significantly shorter than that of synthetic PTFE-based TENGs (>1,000,000 cycles with <5–20 µm wear depth). Although self-healing variants employing dynamic bonds or supramolecular interactions extend durability, their operational lifetimes remain limited to approximately 4500 cycles [49].

The fundamental design-level conflicts associated with performance–sustainability trade-offs make technology maturation and commercialization challenging. Raw biomass offers the lowest cost (USD 0.01–0.10/kg feedstock) and the smallest environmental footprint (near-zero processing energy), but it suffers from poor reproducibility (25–45% output variation), low mechanical stability (<50,000 cycles), and high sensitivity to humidity (>60% output loss at 80% RH) [74]. In contrast, chemically refined materials require multiple hazardous processing steps (e.g., NaOH, H_2_SO_4_, NaClO, KOH), involving substantial chemical consumption, energy input, and waste generation. Life cycle assessment (LCA) studies indicate that the presumed environmental benefits, such as reduced dependence on fossil carbon, lower landfill waste, and diversion of biowaste, can be partially offset by resource-intensive purification processes, particularly when biowaste collection involves long-distance transportation (>100 km) or extensive chemical refinement [179]. To date, comprehensive cradle-to-grave LCAs covering multiple impact categories (global warming potential, cumulative energy demand, water consumption, and toxicity) remain limited, preventing definitive sustainability comparisons. System-level integration and scale-up challenges further hinder industrial viability. Batch laboratory processing (10–100 units) is incompatible with high-throughput manufacturing requirements (>1000 units per hour with <5% defect rates), and cost–benefit analyses remain ambiguous when compared with established energy technologies such as lead–acid batteries (USD 150–250/kWh), lithium ion batteries (USD 200–400/kWh), and utility-scale solar power (USD 0.03–0.08/kWh), placing BW-TENGs at a competitive disadvantage without further technological and economic optimization [74,180,181].

**Figure 11:**
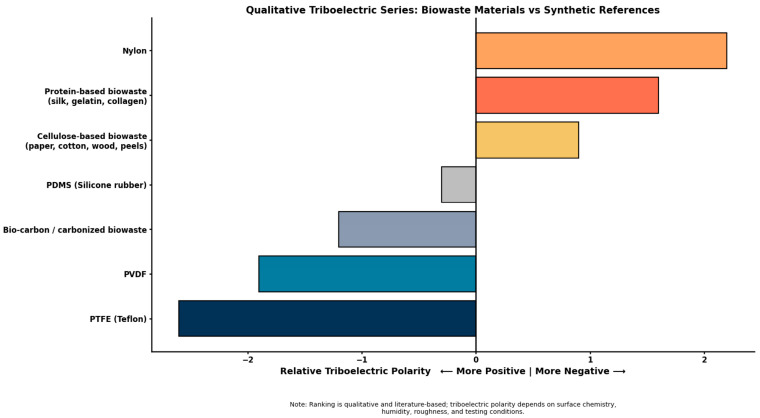
Qualitative triboelectric series comparing representative biowaste-derived material classes with common synthetic reference materials; nylon, PDMS, PVDF, and PTFE [182]; protein-based, cellulose-based, and bio-carbon based [90].

The critical issues in BW-TENG commercialization must be pointed out, with triboelectric polarity limitations directly researched by the comparative triboelectric series in Figure 11. Proteins of biowaste (paper, cotton, membrane, fish scale collagen) are the most strongly positively polarized (electron donors), cellulose-based biomasses (cotton, cellulose-based on wood) are occupied by moderately positive ranges, and the neutral-mid negative range is occupied by bio-carbons (carbonized biowaste). The most favorable BW-TENG designs are the positive cellulose/proteins and negative PTFE/bio-carbon, which can maximize 80 V and optimize the 80 V/cellulose–PTFE hybrids (>300 V). Relative polarity represents the surface charge density measurements in which protein biowastes register 80–120 µC m^−1^ against the PTFE benchmark of −200 µC m^−1^. Plasma functionalization changes cellulose polarity by +15–30% to nylon, reducing constraints observed without jeopardizing sustainability.

## 9. Future Perspectives and Research Directions

The future development of BW-TENG technology will rely on specialized advances in material engineering, process optimization, and system integration [183]. Machine learning and artificial intelligence can offer viable pathways toward faster material discovery and device optimization by minimizing trial-and-error experimentation. Graph neural networks, random forest algorithms, Bayesian optimization, and deep neural networks are data-driven models that can be used to accurately predict triboelectric materials, optimize processing conditions, and inversely design biowaste-derived systems. These techniques, together with extensive experimental feedback, have enabled development timelines on the order of months, shortened prototyping cycles by up to 60%, and enhanced key performance indicators such as voltage output, power density, durability, and environmental stability [184].

Hybrid BW-TENG architectures are required to overcome the inherent drawbacks of single-source TENGs. The introduction of hybrid systems combining TENGs with piezoelectric, electromagnetic, thermoelectric, and solar energy harvesters enables broadband energy capture (0.1–100 Hz), improved robustness under varying conditions, and year-round operation. Multisource hybrid devices have achieved power outputs of 24.6 mW and more than six months of stable, battery-free operation, enabling wireless communication, sensing, and continuous environmental monitoring. BW-TENGs also enable the implementation of multifunctional devices, including integrated energy-sensing platforms, self-powered environmental remediation systems, and biodegradable biomedical implants capable of sensing, stimulation, and on-demand drug delivery. However, large-scale implementation requires international performance standards, standardized testing protocols, and comprehensive cradle-to-grave life cycle analyses. Optimized TENG systems have the potential to reduce CO_2_ emissions by 30–50% and achieve energy payback times as short as 0.5–2 years, supporting conclusions from comparative studies that TENG systems can be considered sustainable.

Short-term commercialization opportunities include large-scale IoT sensor networks, consumer wearables, implantable bioelectronics, and circular bioeconomy integration through waste valorization. Overall, the convergence of AI-enabled optimization, hybrid multifunctional designs, rigorous sustainability evaluation, and standardization positions BW-TENGs as a promising pathway toward scalable, self-powered electronic systems with minimal environmental footprint.

## 10. Conclusions

Triboelectric nanogenerators (BW-TENGs) based on biowaste are of interest as a convergence of sustainable energy generation, waste reuse, and self-powered electronics and align with the tenets of the circular bioeconomy. This article has critically analyzed the principles of triboelectric, types of biowaste materials, processing and surface engineering, architectures, performance, and applications of BW-TENGs. In agricultural, food, marine, medical, pharmaceutical, and plant-based waste streams it has been demonstrated that biowaste resources can be used in applications as effective triboelectric layers, electrodes, and multipurpose components, in most cases having electrical performance equal to conventional synthetic polymer TENGs, and with additional benefits such as biodegradability, biocompatibility, mechanical compliance and low environmental impact. BW-TENGs have shown great potential in a wide field of applications, such as environmental sensing, intelligent agriculture, wearable or implantable bioelectronics, the IoT network, and smart waste management systems. Their natural power generation capability as well as environmental sensing allows them to be implemented in integrated, battery-free systems, especially in distributed, gridless, and resource-limited environments. Moreover, such new concepts as hybrid types of multi-energy harvesters, versatile energy-sensing systems, transient biomedical devices, and circular waste-to-energy systems also testify to the revolutionary potential of BW-TENG technologies. Although this has been achieved, there are still a number of important issues to overcome before BW-TENGs can reach the level of large-scale commercialization. The heterogeneity of materials, poor inter-batch reproducibility, sensitivity to humidity and temperature, shorter mechanical lifetime, and a comparatively low power density relative to optimized synthetic systems are limitations to long-term deployment. Moreover, the absence of standardized testing procedures, full life-cycle analysis and high-scale production routes do not allow for fair benchmarking and economic appraisal. To overcome these limitations, the standardization of materials, the design of greener encapsulation schemes, scalable forms of processing, and the creation of global performance and sustainability standards will be necessary.

## Figures and Tables

**Table 2:** Fruit peel- and eggshell-derived BW-TENG materials: key parameters and performance metrics.

Material/System	Biowaste Source	Device Configuration	Electrical Performance	Key Advantages	Refs.
Lychee peel powder (LPP) TENG	Fresh lychee peels washed with DI water, sun-dried (7 days)	LPP layer (tribopositive)/human skin or PTFE film (tribonegative), contact–separation mode, fabric electrode	V_oc_ = 150–200 V; I_sc_= 2–8 µA; P_max_ = 0.5–2.5 W/m^2^; humidity sensitivity: output maintained across 10–90%RH with 30–40% reduction at high RH	Low-cost simple processing (no chemical treatment); skin-conformal flexibility	[101]
Pomelo peel (citrus) TENG	Pomelo peel (Citrus maxima) washed, dried (60 °C, 24 h)	Pomelo powder/film (tribopositive)/PTFE or Al electrode (tribonegative), contact–separation mode	V_oc_ = 180–250 V; P_peak_ = 255 mW/m^2^; pectin content 13–24 wt%; degree of esterification 63–72% (high-methoxyl pectin)	Naturally porous microstructure; high pectin content providing hydroxyl/carboxyl groups	[103]
Watermelon rind (Citrullus lanatus) TENG	Watermelon rind (white mesocarp tissue) cleaned, dried (60 °C, 12–24 h)	Watermelon rind film (tribopositive)/PTFE film (tribonegative), vertical contact–separation mode, Cu/Al electrodes	V_oc_ = 150–200 V; P_max_= 255 mW/m^2^; continuous operation > 3 months without degradation; cellulose + pectin matrix	Exceptional long-term stability (>90 days); dual cellulose–pectin composition; powered 30 LEDs continuously	[34]
Peanut skin powder (PSP) TENG	Peanut skins (red seed coat) washed, dried (60 °C, 12 h),	PSP layer (tribopositive)/PTFE or PET (tribonegative), stacked configuration with Cu/Al electrodes, controlled air gap	V_oc_ = 162–215 V; I_sc_= 3.5–8 µA; P_max_= 2.2 mW at 20 MΩ; humidity sensitivity 0.8 V/%RH; response/recovery time 4/10 s	Dual functionality: energy harvester + self-powered humidity sensor; cellulose + amino acid (arginine, leucine) composition; chemical tunability via ionic solutions (K_2_SO_4_, NaCl, MgCl_2_)	[104]
Hen eggshell membrane (ESM) TENG	Hen eggshells washed, membranes carefully separated from calcified shell	Hen ESM (tribopositive)/PET or PDMS (tribonegative), multilayer structure with Al/Ag electrodes, contact–separation mode	V_oc_ = 150–200 V; I_sc_ = 0.3–0.4 µA/cm^2^; P~max~ = 10–12 mW; RMS roughness 0.586 µm; collagen matrix (type I, V, X)	Fibrous collagen network; moderate surface roughness; excellent biocompatibility; powered 150–250 LEDs	[67]

**Table 3:** Marine biowaste-derived BW-TENG materials: fish scales and shells—key parameters and performance metrics.

Material/System	Biowaste Source	Device Configuration	Electrical Performance	Key Advantages	Refs.
Fish scale (demineralized) TENG	Raw fish scales (from tilapia, carp, salmon) washed, sequentially treated with 5 wt%	Demineralized fish scale (tribopositive)/PTFE or silicone (tribonegative), vertical contact–separation mode, Cu or Al electrode	V_oc_ = 7.4–39 V; I_sc_ = 0.18–0.6 µA; P_peak_ = 1–5 W/m^2^ (at 50 N force, 3 Hz); preserved lamellar microstructure; collagen fiber diameter 1–5 µm	Hierarchical collagen architecture; intrinsic piezoelectricity (1–5 pC/N)	[108]
Abalone shell powder (ASP) TENG	Raw abalone shells (*Haliotis* spp.) rinsed with deionized water, oven-dried (25 °C, 12 h then 60 °C, 24 h)	ASP layer (tribonegative, CaCO_3_-rich)/TENG with electrodes in custom humidity-controlled chamber, linear motor excitation	V_oc_ = 50–150 V; I_sc_ = 1–8 µA; P_max_ = 5–15 W/m^2^; aragonite content 77.6% (highest among mollusk shells); BET surface area 10–50 m^2^/g	Extraordinarily high aragonite (CaCO_3_ polymorph) content (77.6% vs. 30–60% in oyster shells)	[42]
Oyster shell powder (OSP) TENG	Oyster shells (*Crassostrea* spp.) cleaned, dried (60 °C, 24 h), ground into fine powder (50–150 µm)	OSP layer (tribonegative, CaCO_3_-rich)/TENG structure with Al or Cu electrodes	V_oc_ = 40–120 V; I_sc_ = 1–6 µA; P_max_ = 3–10 W/m^2^; aragonite + calcite mixture (30–60% aragonite); bulk density ~1.8 g/cm^3^	Abundant shellfish-processing waste (>6 million tons/year globally)	[109]
Mollusk shell composite TENG	Mixed mollusk shells (clams, scallops, cockles, mussels) crushed, mixed with epoxy or polydimethylsiloxane (PDMS) binder (20–50 wt%), cured at 60–80 °C	Shell-polymer composite film (tribonegative due to CaCO_3_ dominance)/PTFE or cellulose (tribopositive), contact–separation mode	V_oc_ = 80–200 V; I_sc_ = 2–10 µA; P_max_ = 10–40 W/m^2^; improved mechanical properties vs. brittle pure shells	Enhanced mechanical robustness through polymer binder	[110]

**Table 4:** Medical and pharmaceutical solid waste BW-TENG materials: key parameters and performance metrics.

Material/System	Biowaste Source	Device Configuration	Electrical Performance	Key Advantages	Refs.
Waste X-ray film TENG	Discarded X-ray sheets (polyester/polyethylene terephthalate substrate + silver halide gelatin photosensitive layer)	X-ray film layer (tribonegative, semi-crystalline PET)/silicone rubber or aluminum (tribopositive)	V_oc_ = 110–150 V; I_sc_ = 3–8 µA; P_max_ = 1.39–2.5 W/m^2^ (9 N force, 3 Hz);	Abundant medical imaging waste (>650 million tons polymer waste accumulated globally)	[113]
Expired paracetamol (acetaminophen) TENG	Expired pharmaceutical tablets	Paracetamol film (tribopositive, amide + hydroxyl functional groups)/PTFE or aluminum (tribonegative), vertical contact–separation mode, Cu electrodes	V_oc_ = 561 V; I_sc_= 53 µA; P_max_ = 163 mW (at 20–40 MΩ optimal load); D = 4.0 cm^2^ voltage density: 140 V/cm^2^	High voltage output due to favorable triboelectric polarity; abundant feedstock	[114]
COVID-19 medical waste TENG	Discarded medical protective equipment (PPE) from pandemic	Medical waste polymer layer (tribopositive: cellulose fiber in masks + polypropylene)/PTFE	V_oc_ = 80–200 V; I_sc_= 2–12 µA; P_max_ = 5–20 W/m^2^; multi-layer structure exploits fiber reinforcement from surgical masks	Addresses massive pandemic-era medical waste accumulation (>1 million tons excess PPE globally)	[111]
Medical plastic waste (saline bottles, PVC tubing) TENG	Discarded single-use medical plastics (polyvinyl chloride saline bottles, PVC tubing, polystyrene packaging)	Medical plastic layer (tribopositive: PVC, polystyrene, polyethylene)/PTFE	V_oc_ = 100–250 V; I_sc_ = 3–15 µA; P_max_ = 1.46–8.78 W/m^2^; semi-crystalline polymer structure (crystallinity index 20–60% depending on plastic type)	Abundant medical-sector plastic waste (>5.9 million tons annually worldwide)	[112]
Activated Carbon from Medical Waste	Medical waste (mixed drugs)	Activated medical waste-derived carbon (conductive electrode material)	BET surface area 985–1387 m^2^/g; Electrical conductivity 0.1–10 S/cm; specific capacitance 100–400 F/g; porosity 75–90% pore size distribution micropores < 2 nm (~75% of total pores)	Dual valorization: pharmaceutical waste to electrode + energy storage	[115]

## Data Availability

No new data were created or analyzed in this study. Data sharing is not applicable to this article.
